# Quantitative evaluation of DNA damage repair dynamics to elucidate predictors of autism *vs*. cancer in individuals with germline *PTEN* variants

**DOI:** 10.1371/journal.pcbi.1012449

**Published:** 2024-10-02

**Authors:** Ruipeng Wei, Masahiro Hitomi, Tammy Sadler, Lamis Yehia, Daniela Calvetti, Jacob Scott, Charis Eng

**Affiliations:** 1 Genomic Medicine Institute, Lerner Research Institute, Cleveland Clinic, Cleveland, Ohio, United States of America; 2 Department of Nutrition and Systems Biology and Bioinformatics Program, Case Western Reserve University School of Medicine, Cleveland, Ohio, United States of America; 3 Translational Hematology & Oncology Research, Cleveland Clinic, Cleveland, Ohio, United States of America; 4 Department of Mathematics, Applied Mathematics, and Statistics, Case Western Reserve University College of Arts and Sciences, Cleveland, Ohio, United States of America; 5 Department of Radiation Oncology, Cleveland Clinic, Cleveland, Ohio, United States of America; 6 Taussig Cancer Institute, Cleveland Clinic, Cleveland, Ohio, United States of America; 7 Center for Personalized Genetic Healthcare, Medical Specialties Institute, Cleveland Clinic, United States of America; 8 Department of Genetics and Genome Sciences, Case Western Reserve University School of Medicine, Cleveland, Ohio, United States of America; 9 Germline High Risk Cancer Focus Group, Case Comprehensive Cancer Center, Case Western Reserve University, Cleveland, Ohio, United States of America; Max Planck Institute for Evolutionary Biology, GERMANY

## Abstract

Persons with germline variants in the tumor suppressor gene phosphatase and tensin homolog, *PTEN*, are molecularly diagnosed with PTEN hamartoma tumor syndrome (PHTS). PHTS confers high risks of specific malignancies, and up to 23% of the patients are diagnosed with autism spectrum disorder (ASD) and/or developmental delay (DD). The accurate prediction of these two seemingly disparate phenotypes (cancer *vs*. ASD/DD) for PHTS at the individual level remains elusive despite the available statistical prevalence of specific phenotypes of the syndrome at the population level. The pleiotropy of the syndrome may, in part, be due to the alterations of the key multi-functions of PTEN. Maintenance of genome integrity is one of the key biological functions of PTEN, but no integrative studies have been conducted to quantify the DNA damage response (DDR) in individuals with PHTS and to relate to phenotypes and genotypes. In this study, we used 43 PHTS patient-derived lymphoblastoid cell lines (LCLs) to investigate the associations between DDR and *PTEN* genotypes and/or clinical phenotypes ASD/DD *vs*. cancer. The dynamics of DDR of γ-irradiated LCLs were analyzed using the exponential decay mathematical model to fit temporal changes in γH2AX levels which report the degree of DNA damage. We found that *PTEN* nonsense variants are associated with less efficient DNA damage repair ability resulting in higher DNA damage levels at 24 hours after irradiation compared to *PTEN* missense variants. Regarding PHTS phenotypes, LCLs from PHTS individuals with ASD/DD showed faster DNA damage repairing rate than those from patients without ASD/DD or cancer. We also applied the reaction-diffusion partial differential equation (PDE) mathematical model, a cell growth model with a DNA damage term, to accurately describe the DDR process in the LCLs. For each LCL, we can derive parameters of the PDE. Then we averaged the numerical results by PHTS phenotypes. By performing simple subtraction of two subgroup average results, we found that PHTS-ASD/DD is associated with higher live cell density at lower DNA damage level but lower cell density level at higher DNA damage level compared to LCLs from individuals with PHTS-cancer and PHTS-neither.

## Author summary

*PTEN* is a shared risk gene for both cancer and autism spectrum disorder (ASD) and/or developmental delay (DD). Accurate prediction of the clinical phenotypes of each PTEN hamartoma tumor syndrome (PHTS) individual is still not feasible, though population-level risks of ASD/DD and specific component cancers, including breast cancer and thyroid cancer, have been estimated within PHTS cohorts. In this study we provide insights that PHTS-ASD/DD is associated with faster DNA damage repair rate (half-life of phosphorylated H2A histone family member X, γH2AX, signal over time) and LCLs derived from individuals with PHTS-cancer are related with susceptibility to γ irradiation. PHTS-ASD/DD also showed a higher live cell density when DNA damage was at a low level, and lower live cell density at high DNA damage levels compared to the other PHTS phenotypes. Finally, we provide an innovative way to describe DNA damage response (DDR) in a quantitative and integrative fashion, which is applicable to any type of cells.

## Introduction

Phosphatase and TENsin homolog deleted on chromosome TEN (*PTEN*) encodes a tumor suppressor whose canonical function is dephosphorylation of the growth promoting lipid messenger phosphatidylinositol 3,4,5-trisphosphate (PIP3), thus downregulating phosphoinositol-3-kinase (PI3K)/ threonine-protein kinase (AKT) pathway activity and suppressing cell growth [1–4]. However, PTEN also has other functions, so called “non-canonical functions”, including maintenance of genomic stability [1–4]. PTEN dysfunction at the germline level can cause a wide range of phenotypes including macrocephaly, benign overgrowths and malignant neoplasia, metabolic alterations, and neurodevelopmental disorders (NDD), such as autism spectrum disorder (ASD) and developmental delay (DD) [5]. All these phenotypes, when associated with germline *PTEN* alterations, fall into an umbrella term called PTEN hamartoma tumor syndrome (PHTS). Two prevalent yet disparate clinical phenotypes observed in individuals with PHTS are cancer and ASD/DD. Indeed, *PTEN* is a shared risk gene for cancer and ASD [6,7]. Individuals with PHTS not only have elevated lifetime risks of cancer, but also, 23% are diagnosed with ASD/DD as well [5,8,9]. Although rare, ASD/DD and cancer are not always mutually exclusive phenotypes in PHTS. Additionally, *PTEN* genotype alone is not sufficient to dictate phenotype: in some families, a parent and a child who have the same germline *PTEN* mutation have disparate clinical phenotypes such as cancer (without ASD/DD) *vs*. ASD/DD (without cancer). Previous studies have attempted to identify *PTEN* genotype-phenotype associations relevant to specific *PTEN* mutation types and/or PTEN activity profiles; however, these data have been inconsistent [10,11]. Indeed, we can accurately predict the prior probability of organ-specific cancers in a cohort of individuals with PHTS (*vs*. any PHTS individual), and we can do equally well in predicting ASD/DD prior probability as 23% of children with PHTS at the group level [7,8,12]. However, it is challenging to predict with high accuracy which individual (*vs*. group) will develop cancer and/or ASD/DD when they carry a germline *PTEN* variant.

Earlier studies have shown that higher germline copy number variations (CNVs) are associated with both sporadic/idiopathic ASD and cancer [2,13–20]. Recently, we found that patients with PHTS-ASD/DD have a higher genome-wide burden of CNVs than individuals from the PHTS-cancer group [19]. One source of CNV generation is failed homologous recombination (HR) during the DNA damage repair process [14,19,21].

PTEN plays an important role in repairing DNA damage. AKT, which is downregulated by PTEN, has been shown to prevent the translocation of BReast CAncer gene 1 (BRCA1) to DNA damage sites [18,22]. BRCA1 participates in HR and non-homologous end-joining, the two predominant repair pathways for DNA double strand breaks (DSB) [18,22]. PTEN also contributes to DNA DSB repair by regulating RAD51 Recombinase (RAD51) transcription [13]. Relatedly, PTEN null cells have decreased RAD51 expression and are sensitive to DNA damage [17]. PTEN’s interaction with the centromere protein CENP-C is essential for chromosome stability. Disruption of PTEN therefore causes centromere breakage and chromosomal translocations [17]. PTEN also interacts with histones and non-histone chromatin architectural proteins to maintain a condensed chromatin structure [2]. Additionally, specific *PTEN* variants, including K254R and Y68H, have been shown to be associated with DNA damage repair defects [1,15]. Thus, we postulate that the dynamic DNA damage response (DDR) in a background of germline *PTEN* variants may serve as a potential marker to predict PHTS clinical phenotypes at the individual level.

There are many mathematical models to describe cell growth, for example, logistic growth function and linear function [23]. However, they typically describe cell growth in one dimension (i.e., time) [24,25]. Meanwhile, previous studies also describe the cellular DNA damage repair dynamics in the time course [26,27]. Currently, there is no mathematical model that quantifies cell growth dynamics over time in relation to DNA damage. To accurately predict PHTS clinical phenotypes (cancer vs. ASD), we would like to explore the quantitive association between PTEN genotype and/or PHTS clinical phenotypes (cancer vs. ASD) and the genomic integrity phenotype at the same time. The Reaction-diffusion Partial Differential Equation (PDE) is a well-suited approach to dynamically quantify PTEN’s function in the DNA damage response (DDR), including DNA damage and cell growth. In other words, we can consider both DNA damage and cell growth terms into this model. Via this combination, the model can quantify how cell proliferation varies within the highly heterogenous PHTS cohort. At the same time, the model also can explain how cell proliferation is affected by DNA damage and repair dynamics. Accordingly, we aim to quantify the association between DDR, germline *PTEN* variants and/or PHTS clinical phenotypes (ASD/DD *vs*. cancer) using this reaction diffusion PDE mathematical model through this study.

## Results

### DNA damage repair dynamics of LCLs with various PTEN genotypes

**Fig 1:**
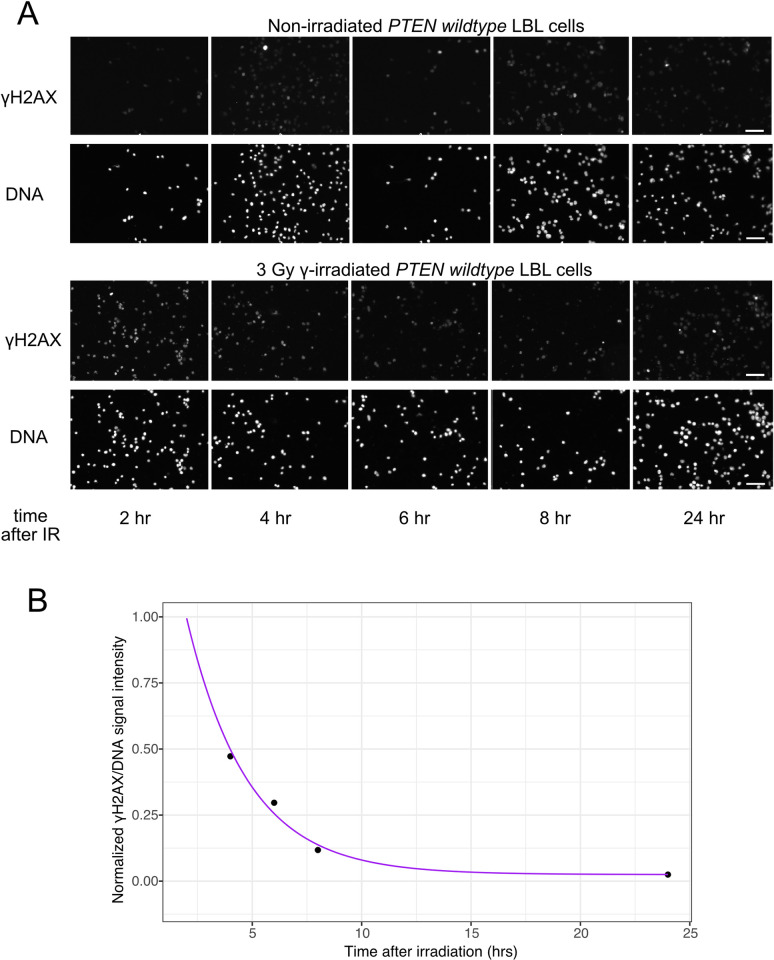
Quantification and normalization of DNA damage repair kinetics of lymphoblastoid cell lines (LCLs) derived from individuals with PHTS-ASD/DD, PHTS-cancer, and the healthy donor. A. Representative images of γH2AX and DNA immunofluorescence. B. A representative DNA damage exponential decay curve deduced from the normalized averages of fluorescence intensities of γH2AX in individual cells.

Former studies have shown that PTEN’s C2 region and K254 site are associated with genomic integrity [1,17]. Therefore, we hypothesized that LCLs derived from individuals with PHTS and harboring truncated C2 and/or variants on and close to the K254 site may inefficiently repair DNA damage. To address this hypothesis, we performed time-course γH2AX immunofluorescence experiments to capture LCLs’ DNA damage level change after 3 Gy irradiation treatment. (Fig 1A) We used the difference between paired treated and untreated γH2AX/DNA intensity to represent the DNA damage levels. To normalize the DNA damage level across batches, we used the PTEN WT LCL as an internal control in every batch. To minimize batch effects, DNA damage levels from all batches were normalized by the control LCL’s DNA damage level at 2 hrs after IR. (Fig 1B)

PTEN is a guardian of genomic integrity. To examine the association between PTEN genotypes and DNA damage repair ability, we collected LCLs from individuals with PHTS and various *PTEN* genotypes. To specifically explored which *PTEN* genotype is associated with lack of DNA damage repair ability, we selected PHTS patient derived LCLs with *PTEN* variants based on the reported disrupted DDR caused by PTEN mutations. A former study indicated that cells with PTEN C2 region truncation have a limited ability to maintain genome integrity [1]. We examined DDR of PHTS individual-derived LCLs with nonsense mutations generating PTEN C2 region full (p.Try88*, p.Arg130*) or partial (p.Arg335*) truncation. Another study previously showed that mutation on PTEN SUMOylation site (p.Lys254) imparted the cells with more sensitivity to irradiation and deficiency to repair DNA damage [1]. In our cohort, we selected two LCLs with PTEN missense mutations at the SUMOylation site (p.Lys254Thr) and two LCLs with missense mutations that are in the proximity to the SUMOylation site (p.Asp252Val, and p.Asp252Gly) to test their effects on DDR. Nuclear PTEN has been proven to be essential for maintaining genome integrity. Our lab found PTEN accumulates within the nucleus in cells harboring the PTEN Try68His variant [28]. Thus, we included LCLs with PTEN Try68His variant for our DDR assay. In addition to these mutants with reported DDR deficits, we also included PTEN Arg173Cys due to its prevalence as a pathogenic variant in our PHTS cohort, irrespective of phenotypes. Additionally, we included pathogenic and/or likely-pathogenic variants on PTEN splicing sites or the promoter region for being frequently observed in our cohort.

**Fig 2:**
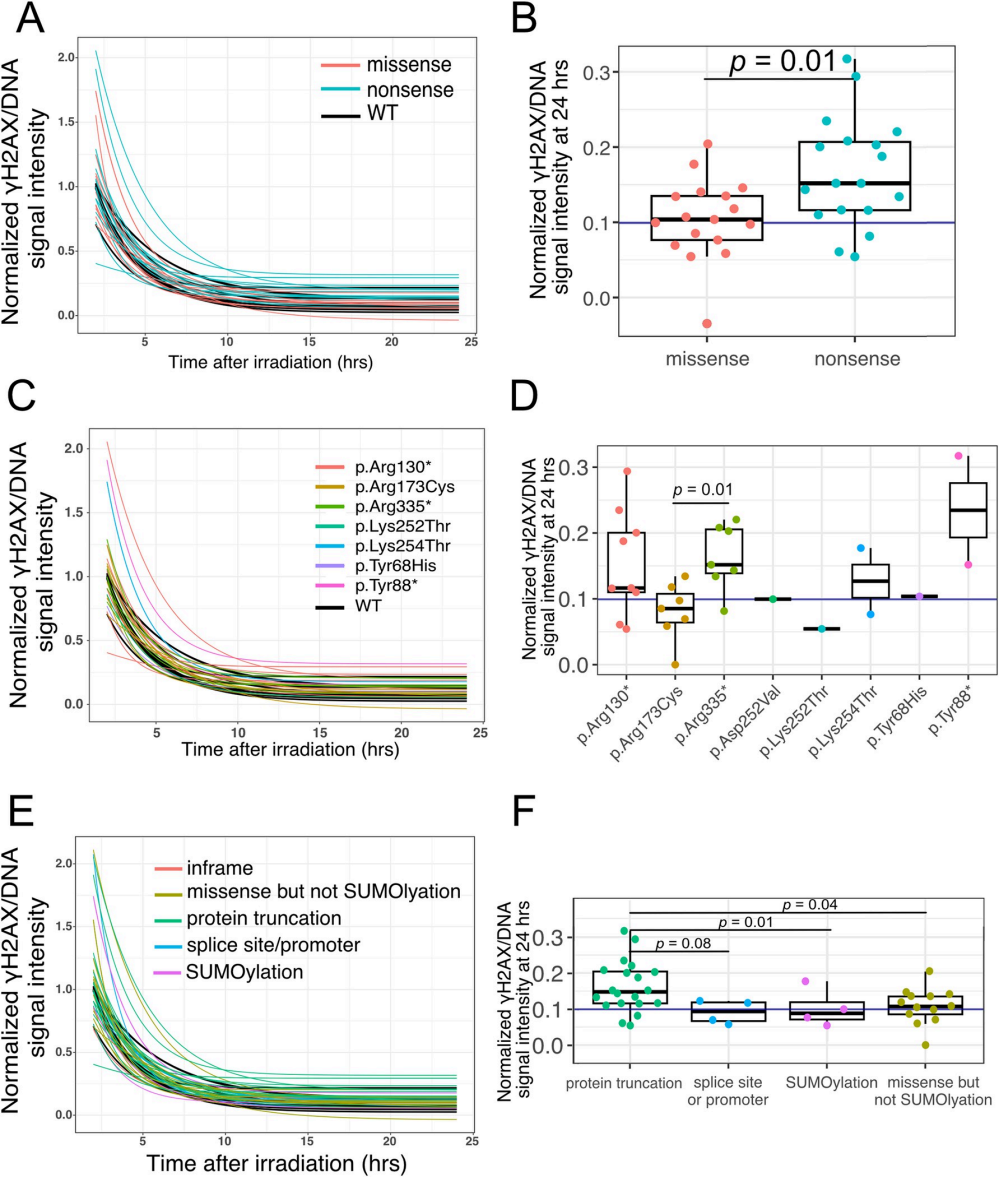
Comparison of DNA repairing capacity of LCLs with various *PTEN* genotypes. A. DNA repair kinetics after 3 Gy irradiation are depicted by normalized DNA damage exponential decay curves for 43 LCLs color-coded according to their PTEN variant effect types. B. Box plot illustrates the distribution of DNA damage levels (normalized γH2AX/DNA signal intensity) at 24 hrs after irradiation for LCLs of indicated PTEN variants. The purple line represents the average DNA damage level of wildtype (WT) LCL replicates. Wilcoxon statistical analysis detected that R335* residual levels of DNA damage were significantly higher than those observed in R173C and wild-type. C. Normalized DNA damage exponential decay model of 43 PHTS LCLs colored by their PTEN variant category. Inframe indicates the LCL contains PTEN c.39_41delAAG; SUMOylation indicates LCLs which contain PTEN p.Lys254Thr or p.Asp252Val variants; protein truncation/loss indicate LCLs contain PTEN variants which cause PTEN truncation, deletion or loss; missense but not SUMOylation indicates LCLs contain PTEN missense variants but not the p.Lys254Thr or p.Asp252Va variants; splice site or promoter indicates LCLs contain splice site PTEN variants or those within the promoter. D. Box plot of DNA damage level (normalized γH2AX/DNA signal intensity) at 24 hrs after irradiation clustered by different PTEN variant categories described in panel G. The purple line represents the average DNA damage level of wildtype LCL replicates. LCLs carrying truncated variants or large deletion (c.80-?_164+?del (E2 del) have significant higher residual DNA damage levels than other subgroups, splice site/promoter, SUMOylation, missense, but not SUMOylation, protein truncation. E. Normalized DNA damage exponential decay model of 43 PHTS LCLs colored by their PTEN variant effect. F. Box plots of DNA damage levels (normalized γH2AX/DNA signal intensity) at 24 hrs after irradiation categorized by PTEN variant effect. The horizontal purple line represents the average DNA damage level of wildtype LCL replicates. Wilcoxon statistical analysis detected significant DNA damage residuals at 24 hrs after irradiation between LCLs carrying nonsense PTEN variants and missense PTEN variants.

We compared the difference of DNA damage residuals at 24 hours following IR among these selected LCLs. We found that LCLs with nonsense *PTEN* variants have statistically significantly higher DNA damage residual levels than those harboring *PTEN* missense variants (*p* = 0.01) (Fig 2A and 2B). To determine which *PTEN* genotype correlates with higher DNA damage repair efficiency, we performed pairwise comparisons of DNA damage residual at 24hrs after IR among PTEN p.Arg130*, p.Arg173Cys, and p.Arg335* variants. Specifically, we discovered that LCLs with the PTEN Arg335* variant causing PTEN C2 domain partial deletion have a higher DNA damage level at 24 hrs after IR than those with PTEN Arg173Cys (*p* = 0.01) (Fig 2C and 2D). Because missense group is associated with more efficient DNA damage repair, while SUMOylation is opposite. We excluded LCLs with SUMOylation variants (p.Lys254Thr, p.Asp252Val and p.Asp252Gly, which are on or close to the SUMOylation site, Lys254) from the missense group and make these four samples as a new subgroup, SUMOylation. In this way, we can observe whether variants located on or close to SUMOylation sites are associated with DNA damage repair inefficiency in our cohort, and whether other missense variants are still associated with efficient DNA damage repair. We included LCLs with variants affecting splice sites (*PTEN* c.802-2A>G, c.210-1G>A, c.-1170C>T, and c.634+5G>C), which are pathogenic or likely pathogenic variants [29] as well. Relatedly, we combined two samples with variants (PTEN p.Tyr27Cysfs*44 and p.Cys211fs) which cause PTEN truncation, and PTEN nonsense groups together as protein truncation group. In these comparisons, we still found that those LCLs with protein truncation have statistical higher DNA damage residuals at 24 hrs than the missense but not the SUMOylation group (*p* = 0.04); and the higher trend than comparing protein truncation (*p* = 0.01) (Fig 2E and 2F). In summary, we show that LCLs with missense *PTEN* variants have less DNA damage residual at 24 hrs after irradiation than *PTEN* nonsense variants; LCLs with variants on SUMOylation sites trend towards the least DNA residual at 24 hrs after IR compared with other missense variants, variants on splicing sites and variants causing PTEN truncation.

### DNA damage repair dynamics of LCLs from PHTS phenotype subcategories

Our previous study indicated that PHTS-ASD/DD has higher genome-wide copy number variations (CNVs), which include pathogenic and likely-pathogenic CNVs when compared to PHTS individuals without ASD/DD [19]. One source of CNV generation is unsuccessful homologous recombination which is a key mechanism of DNA double strand break (DSB) repair [14,21]. Hence, we hypothesized that LCLs derived from individuals with PHTS-ASD/DD are associated with a slower DNA damage repair rate and/or insufficient DNA damage repair.

**Fig 3:**
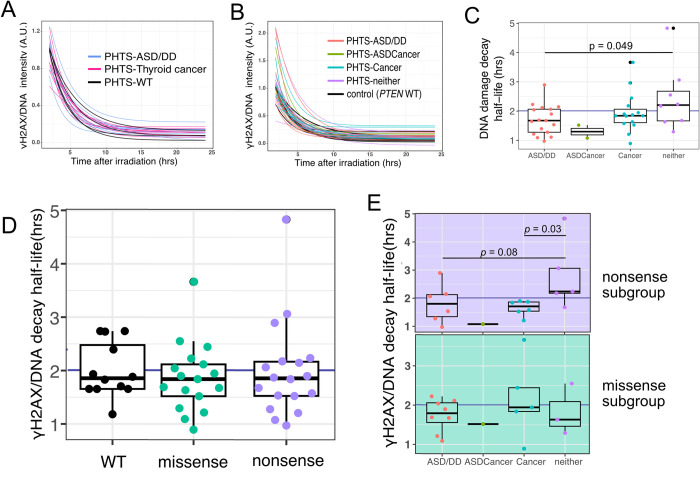
DNA damage repairing rate of PHTS samples. A. Normalized DNA damage decay curves of LCLs derived from 20 age and sex matched patients with PHTS-ASD/DD, PHTS-cancer, and from a *PTEN*-wild-type donor with several biological replicates from every batch. B. Normalized DNA damage repairing exponential decay model of 43 PHTS LCLs colored by PHTS phenotype. C. Box plots of the half-lives of DNA damage deduced from the exponential decay model, clustered by phenotypes from 43 PHTS LCLs. Detected by Wilcoxon statistical test, PHTS-neither related LCLs have higher DNA damage decay half-life than PHTS-ASD/DD. D. Box plots of the half-lives of DNA damage deduced from the exponential decay model of LCLs derived from patients with PTEN nonsense and missense variants, as well as wild-type *PTEN* donor biological replicates. E. Upper panel is a box plot of the exponential decay model half-life DNA damage repair capacity after irradiation of LCLs harboring PTEN nonsense variants, with samples clustered by phenotype. Detected by Wilcoxon statistical test, PHTS-neither related LCLs have higher DNA damage decay half-life than PHTS-ASD/DD. The lower panel is a box plot of the exponential decay model half-life DNA damage repair capacity after irradiation of LCLs harboring PTEN missense variants, with samples clustered by phenotype.

When comparing DNA damage repair curves from age and sex matched PHTS-ASD/DD and PHTS-thyroid cancer groups, we could observe that PHTS-thyroid cancer group has more homogenous DNA damage repairing performance than PHTS-ASD/DD group (Fig 3A). LCLs derived from individuals with PHTS-ASD/DD have shorter DNA damage decay half-life than those in the PHTS-neither group (p = 0.049) (Fig 3B and 3C). Within the LCLs harboring nonsense variants, we observed the trend that PHTS-neither has longer half-life of DNA damage repair curve than the PHTS-Cancer group (p = 0.03) (Fig 3D and 3E).

### Change of LCL growth rate after irradiation is associated with PHTS phenotype

PTEN’s canonical function regulates cell proliferation via antagonizing the PI3K-AKT pathway [30,31]. Notably, PTEN also serves as a guardian of genome integrity. Specific *PTEN* mutations such as K254R have been reported to associate with inefficient DNA damage repair [1]. We therefore asked, under γ irradiation (IR), a DNA damage inducer, how cell growth rate change is associated with PHTS phenotypes or *PTEN* genotypes.

We performed experiments to detect maximum cell growth rate and carrying capacity without irradiation treatment. Then we compared the maximum cell growth rate and carrying capacity by their corresponding PHTS phenotype subcategories or *PTEN* genotypes. While Wilcoxon test did not detect any statistical significance in both parameters between any of the phenotypic or genotypic subgroups (S1 and S2 Figs). To increase analytical power, we also performed statistical testing between *PTEN* nonsense and *PTEN* missense groups, *PTEN* variants within coding regions *vs*. *PTEN* variants within non-coding regions, and the PHTS-ASD/DD group *vs*. the PHTS-noASD/DD group, as this grouping increased the sample number per category. However, we still observed no differences. (S3–S8 Figs)

**Fig 4:**
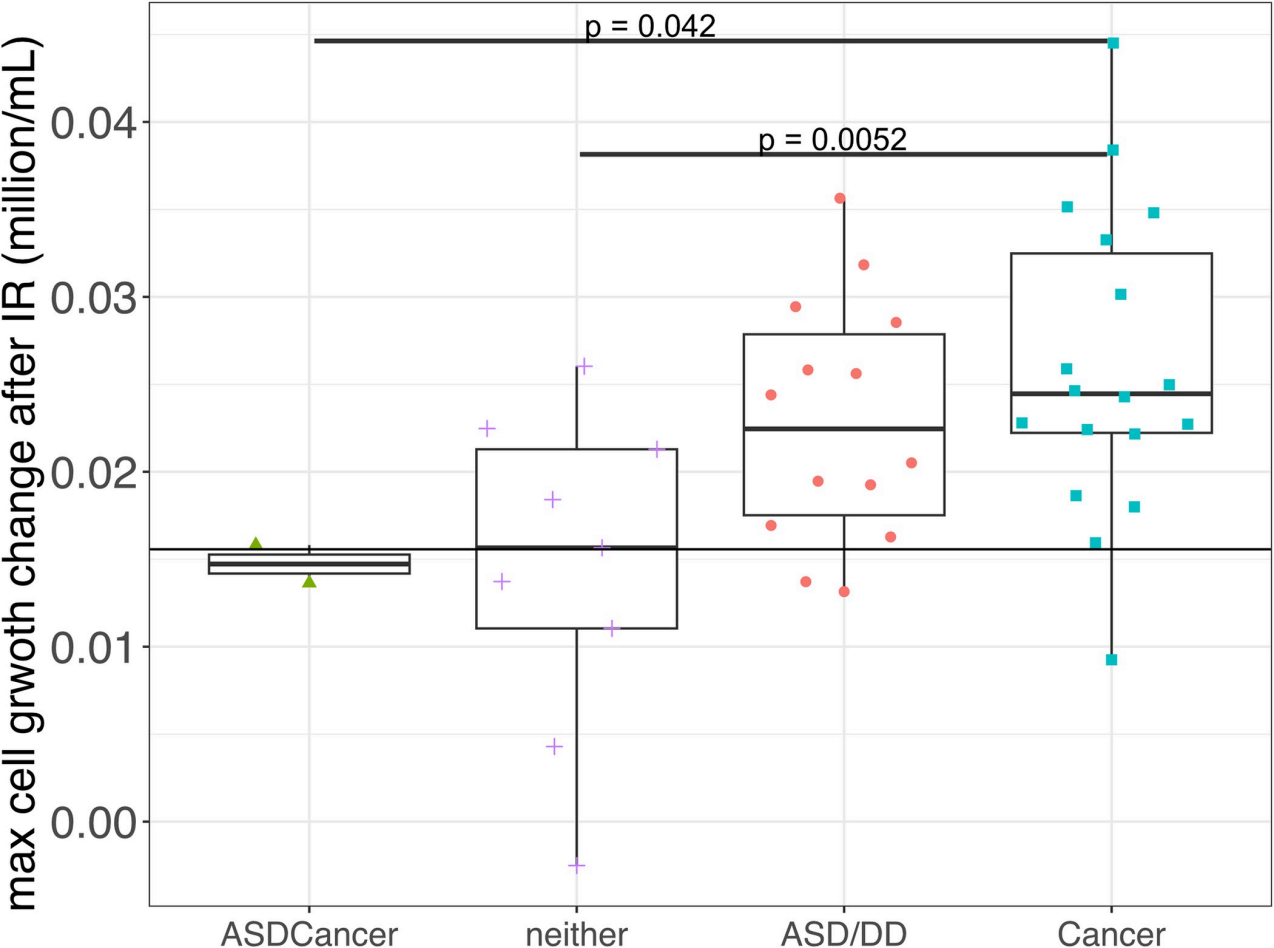
Box plot of LCLs’ difference of maximum growth rate with and without of different 3 Gy irradiation categorized by PHTS phenotypes. Detected by Wilcoxon statistical test, PHTS-cancer related LCLs have higher maximum cell growth rate change than PHTS-ASDCancer and PHTS-neither.

We also conducted experiments to capture the maximum growth rate of the 43 LCLs after 3 Gy IR. We did not observe any significant difference when comparing the rate between different PHTS phenotypes and *PTEN* genotypes (S9–S11 Figs). We then calculated the cell growth rate change with and without IR by calculating the difference of the maximum cell growth rate treated with 0 Gy and treated with 3 Gy IR for all 43 LCLs (S12–S15 Figs). We found that LCLs derived from individuals with PHTS-cancer have the highest cell growth rate change among all four phenotype groups, with statistical significance when comparing with PHTS-neither (p = 0.0052). We also observed that PHTS-ASDCancer subgroup has a trend of having lower cell growth rate change than PHTS-Cancer subgroup (Fig 4).

### PHTS phenotype is associated with LCLs DNA damage response

**Fig 5:**
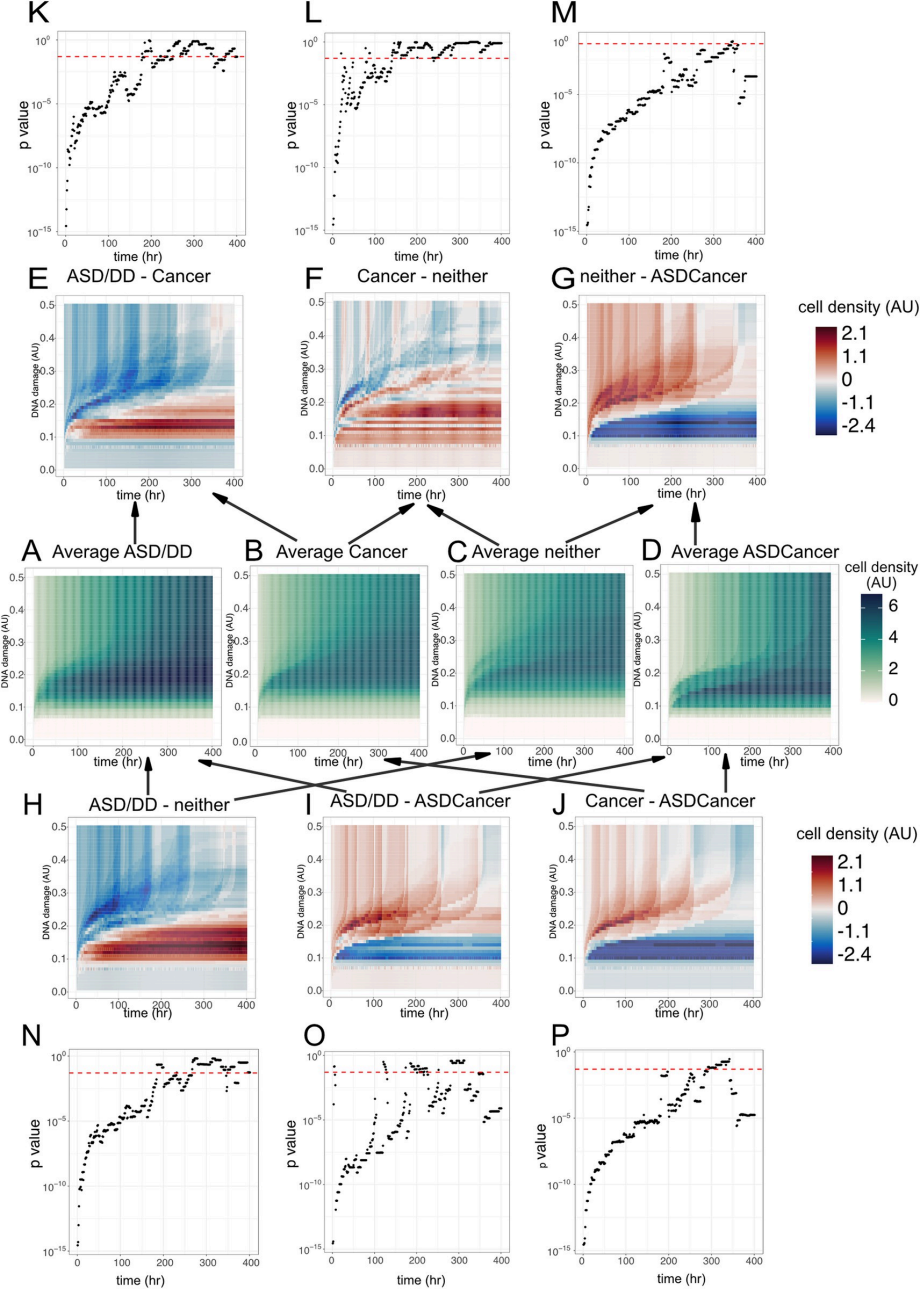
Heatmaps of numerical results generated from the reaction-diffusion partial differential model, subcategorizing by phenotypes and dot plots of the statistical comparison results between different numerical results of phenotypes. A-D: Average numerical result of LCLs source from PHTS-ASD/DD individuals, PHTS-cancer individuals, PHTS-neither individuals, PHTS-ASDCancer individuals. Color represents the cell density, the deeper the higher. E-G: Heatmap of the difference between average numerical result of LCLs source from PHTS-ASD/DD individuals and average numerical result of LCLs source from PHTS-cancer, PHTS-cancer individuals and average numerical result of LCLs source from PHTS-neither, PHTS-neither individuals and average numerical result of LCLs source from PHTS-ASDCancer, respectively. H-J: Heatmap of the difference between average numerical result of LCLs source from PHTS-ASD/DD individuals and average numerical result of LCLs source from PHTS-neither, PHTS-ASD/DD and PHTS-ASDCancer, PHTS-Cancer and PHTS-ASDCancer respectively. K-M: Dot plots of the p values generated by Kolmogorov–Smirnov test between average numerical result of LCLs source from PHTS-ASD/DD individuals and average numerical result of LCLs source from PHTS-cancer, PHTS-cancer individuals and average numerical result of LCLs source from PHTS-neither, PHTS-neither individuals and average numerical result of LCLs source from PHTS-ASDCancer, respectively. Each black dot represents the p value generated at the corresponding time point. Red dashed line represents p value = 0.05. N-P: Dot plots of the p values generated by Kolmogorov–Smirnov test between average numerical result of LCLs source from PHTS-ASD/DD individuals and average numerical result of LCLs source from PHTS-neither, PHTS-ASD/DD and PHTS-ASDCancer, PHTS-Cancer and PHTS-ASDCancer respectively. Each black dot represents the p value generated at the corresponding time point. Red dashed line represents p value = 0.05.

We then applied the DNA damage and cell growth data into the modified reaction-diffusion PDE to generate the integrated, quantified DDR results. Thereafter, we visualized the average numerical results of LCLs categorized by their corresponding PHTS phenotype (Fig 5A–5D). By calculating the difference of average numerical results of two phenotypes, we can directly compare the DDR between different phenotypes (Fig 5E–5J). Differences are depicted by cooler or warmer colors on these heatmaps. To find the difference of DDR between the distinct PHTS phenotypes, we also compared the paired average cell density in different phenotype subgroups at the fixed timepoints by Kolmogorov–Smirnov test (Fig 5K–5P). LCLs show similar cell density regardless of the phenotype when DNA damage is less than 0.1. We can conclude that LCLs derived from individuals with PHTS-ASD/DD have higher live cell density than those from PHTS-cancer and PHTS-neither when the DNA damage level range is from 0.15 to 0.25 and the post irradiation time is from 50 hrs to 400 hrs. However, when DNA damage level is above 0.25, cell density of PHTS-ASD/DD subgroup is less than PHTS-neither and PHTS-cancer subgroup. While when PHTS-ASD/DD is compared with PHTS-ASDCancer group, at specific time points, ASD/DD group is associated with lower cell densities at a lower DNA damage range (0.1–0.18), higher cell densities at a higher DNA damage range (0.18–0.5) except when DNA damage level was above 0.4 and post irradiation time beyond 350 hrs. When the post irradiation time is from 0 to around 200 hrs, the average numerical results between ASD/DD and Cancer subgroups have statistically significant difference.

For PHTS-cancer *vs*. PHTS-neither, we found the difference to be more complex than other pairs. The trend is at a fixed timepoint, PHTS-cancer group has higher relative cell density at lower DNA damage range (~0.1–0.3), but higher cell density at higher DNA damage range (~0.3–0.5). While within the lower DNA damage area PHTS-cancer shows lower cell density at some time points (t = 20, 80, 150 hours). When time is from 0 to around 150 hrs, the average numerical results between ASD/DD and Cancer subgroups have statistically significant difference.

When comparing PHTS-cancer with PHTS-ASDCancer, we found that at lower DNA damage levels (0.1–0.2) or after 300hrs after IR, LCLs derived from individuals with cancer have higher cell density, otherwise PHTS-ASDCancer group has higher cell density. Similarly, when we calculate the difference between PHTS-neither and PHTS-ASDCancer, at lower DNA damage levels (0.1–0.2) or at 350 hrs after IR, LCLs derived from individuals with neither ASD nor cancer phenotype have higher cell density. When time is from 0 to around 300 hrs, the average numerical results between ASD/DD and Cancer subgroups have statistically significant difference.

## Discussion

In this study, we quantitively investigated the DNA damage repair and cell growth of LCLs derived from individuals with PHTS. We also used a PDE model to quantify the DNA damage response process while considering both DNA damage and cell growth together. We devised a series of experiments to measure the growth of LCLs, which were subsequently quantified using the logistic growth equation model. To clarify, through these experiments, we ascertained the values of exponential growth rate and maximum carrying capacity. Following this, we performed a time-course IF for γH2AX to monitor the DNA damage repair process in LCLs post γ IR treatment. These quantified results were then incorporated into the exponential decay model, enabling us to derive values for A, B, and C. By merging the cell growth and DNA damage repair models, we integrated them into the reaction-diffusion PDE, which allows us to characterize the temporal changes in cell density within the context of DNA damage.

The DNA damage observations may contribute to addressing the clinical challenge of predicting ASD/DD and/or cancer phenotypes in individuals with PHTS. The application of the reaction-diffusion model provides an innovative way to quantitatively describe DDR levels in cells, which includes integrating cell growth and DNA damage together. Also, this method can be easily applied to DDR of other cell types.

PTEN’s C2 domain has been reported to be associated with genome integrity via physical interaction with CENP-C to maintain centromere stability and regulation of Rad51 to control DSB repair [17]. In this study, we examined DNA damage response of patient derived LCLs with *PTEN* nonsense mutations that fully (p.Try88*, p.Arg130*) or partially (p.Arg335*) truncate the PTEN C2 region. In the DNA damage repair experiments, we observed that LCLs with these mutations repair DNA damage less efficiently than LCLs with other mutations with intact C2 domain. These findings support the former observation that PTEN, especially its C2 domain, is critical in the DNA repair process.

A previous study also indicated that mutation on K254 of *PTEN* will make cells more sensitive to DNA damage stress and inefficient in DNA damage repair [1]. Such mutation blocks PTEN translocation from the cytoplasm to the nucleus by disabling SUMOylation on the K254 site, which promotes nuclear import of PTEN. PTEN retention in the cytoplasm reduces availability of PTEN to be involved in the steps required to maintain genome integrity such as DNA repair. To test this possibility, we tested DNA damage response of LCLs that have K254T, a mutation in the SUMOylation site, but not the exact same mutation as the former study. We also tested two other LCLs with D252V and D252G mutations that are located close to the 254 SUMOylation site. Contrary to our prediction, we did not find differences between these four as a group and other specific *PTEN* genotypes or *PTEN* variant effect in DNA damage repair. We only found that LCLs with these four mutations have lower DNA damage residuals at 24 hrs after IR than LCLs with truncated PTEN, although this observation was not statistically significant (*p* = 0.1). It is also possible that our sample size was too small to detect differences. Additionally, we cannot draw any conclusion whether the two mutations on PTEN amino acid 252 position have an effect on LCLs’ DNA damage repair due to the limited sample size.

Our previous study found that PHTS-ASD/DD has higher CNV burden than those PHTS individuals without ASD/DD [19]. One source of CNV generation is unsuccessful homologous recombination [14]. In this study, what we observed was that LCLs derived from individuals with PHTS-neither phenotype had slower DNA damage repair rate than PHTS-ASD/DD in all selected samples. Meanwhile, within the subgroups with *PTEN* nonsense variants, PHTS-neither still showed slower DNA damage repair rate than PHTS-cancer and the slower trending of DNA damage repair rate than PHTS-ASD/DD (*p* = 0.08). Also, LCLs derived from PHTS-ASD/DD showed faster repair rate than those derived from PHTS-neither. Considering PHTS-ASD/DD samples are associated with higher CNV, we think the DNA damage repair rate is not associated with the amount of CNVs generated.

We also found that the maximum cell growth rate of samples from the PHTS-Cancer groups were affected most under 3 Gy irradiation treatment compared to LCLs from other PHTS phenotypes with the same treatment. However, we did not find any significant difference in maximum cell growth rate between PHTS phenotype subgroups when samples were not treated with irradiation. We still can conclude that LCLs derived from individuals with PHTS-Cancer are more sensitive to irradiation than PHTS-ASD/DD and PHTS-ASDCancer in the cell growth aspect.

We used the average and the difference of averages to quantitatively compare the samples’ DDR to their PHTS phenotypes (Fig 5). Based on this method, we observed that within 200hrs after irradiation treatment, the cell densities of different PHTS phenotype subgroups are very different, *i*.*e*. *p* values are less than 0.05 via using the Kolmogorov–Smirnov test. But when after 300 hrs of irradiation treatment, *p* values of all paired comparisons are become larger than 0.05, which means at these timepoints, the DDR from different PHTS phenotype subgroups do not have statistical significance. We interpreted this as these PHTS individuals derived LCLs achieved the equilibrium when time over 300hrs after irradiation treatment. This observation indicates that further study may pay more attention to the DNA damage repair process and the cell exponential growth phase rather than the whole DDR process. This method provided a new way to interpret the quantified DNA damage response process and may contribute to the more accurate prediction of PHTS phenotypes. Besides, in the context of the presence of mutations in other DDR genes such *RAD51* or *BRCA1*, it would be interesting to quantitively compare DDR between LCLs with germline *PTEN* variants compared to those with germline variants in these other DDR genes via our PDE model. Indeed, comparing these dynamics with other canonical DDR gene contexts may help elucidate mechanisms in future studies.

PHTS is a rare disordere and it is often difficult to obtain a good sample size to perform statistical analyses even using our patient cohort, which is one of the largest cohorts in the field today. Our sample size of PHTS-ASDCancer phenotype is extremely limited, we only observe some trends but hard to make any conclusion of this phenotype. Additionally, we did observe some trends, for example PHTS-ASD/DD subgroup has faster DNA damage repair rate than other groups when all of the samples carrying PTEN nonsense mutation, (Fig 3E), we still cannot make a solid conclusion due to a small sample size. We hope to do further study with a larger sample size and prove this results. Building an interinstitutional collaborative consortium of data and biobank would help facilitate such studies.

## Materials and methods

### Ethics statement

Individuals diagnosed with PTEN hamartoma tumor syndrome (PHTS) were accrued in accordance with research protocol 8458-PTEN, approved by the Cleveland Clinic Institutional Review Board. To address our research question on phenotype-dependent differences in the non-canonical PTEN signaling pathway, we first selected two age- and sex-matched patient series, one consisting of 10 individuals with PHTS and ASD and/or DD without any personal history of cancer and another comprising 10 individuals with PHTS diagnosed with thyroid cancer. Next, we selected 31 patients who have specific germline *PTEN* mutations to explore genotype-phenotype associations. We studied a total of 43 subjects with 8 overlapping patients between the series. For all selected participants, we reviewed medical records, including clinical genetic testing reports, pedigrees, clinical notes associated with cancer genetics and/or genetic counseling visits, and ASD Diagnostic and Statistical Manual of Mental Disorders (DSM-IV) criteria, where applicable. Written informed consents were obtained from all research participants. We categorized samples into four phenotypes, PHTS-ASD/DD (only ASD/DD), PHTS-Cancer (only cancer), PHTS-ASDCancer (both ASD/DD and cancer), and PHTS-neither (no ASD/DD and no cancer). (Table 1 and Table 2)

**Table 1:** PHTS phenotype and demographic data of research participants.

Group	Explanation	Total	Sex(F/M)	Median Age at Consent (range)
All except WT	All samples with germline *PTEN* variants	43	23/20	32(2–65)
PHTS-ASD/DD	Diagnosed with only ASD/DD	17	8/9	14(2–46)
PHTS-Cancer	Diagnosed with only cancer	15	11/4	25(8–65)
PHTS-ASDCancer	Diagnosed with both ASD/DD and cancer	2	2/0	42(35–49)
PHTS-neither	Diagnosed with neither ASD/DD nor cancer	8	2/6	42.5(13–51)

**Table 2:** *PTEN* genotypic data of research participants.

*PTEN* Genotype	Amino acid change	Sample size	Variant effect	Variant Category	Variant region(coding/non-coding)
c.76A>C	p.Thr26Pro	1	Missense	Missense but not SUMOlyation	Coding
c.103A>G	p.Met35Val	1			
c.202T>C	p.Thr68His	1			
c.209T>C	p.Leu70Pro	1			
c.389G>T	p.Arg130Leu	1			
c.406T>C	p.Cys136Arg	1			
c.517C>T	p.Arg173Cys	7			
c.755A>T	p.Asp252Val	1		SUMOlyation	
c.755A>G	p.Asp252Gly	1			
c.761A>C	p.Lys254Thr	2			
c.39_41delAAG	p.Arg14del	1	Inframe	Inframe	
c.80-?_164+?del (E2 del)	p.(Tyr27Cysfs*44)	1	Deletion	Protein truncation	
c.264T>A	p.Tyr88*	2	Nonsense		
c.388C>T	p.Arg130*	9			
c.1003C>T	p.Arg335*	8			
c.632delG	p.Cys211fs	1	Frameshift truncation		
c.802-2A>G	NA	1	Splice acceptor	Splitce site/promoter	Non-coding
c.210-1G>A		1			
c.-1170C>T		1	5’ UTR		
c.634+5G>C, p.Gly165Ilefs*9		1	Exon skipping/Splice acceptor		
WT	WT	1	NA	WT	NA

The research adhered to the principles of the Declaration of Helsinki and ensured confidentiality and protection of participants’ personal and medical data. The research protocol ensured that there was no discrimination based on race, gender, or socio-economic status. All aspects of the study were designed to minimize any risks to the participants, and no interventions beyond standard clinical practices were introduced. Furthermore, the study had a provision for participant withdrawal at any stage without any consequences to their medical care or rights.

### Lymphoblastoid cell line generation and culture

Lymphoblastoid cell lines (LCLs) were generated from peripheral blood leukocytes of research participants using a standard method (https://www.lerner.ccf.org/genomic-medicine/documents/GMB004%20Lymphoblasts_v2016.pdf) which is routinely used at the Genomic Medicine Biorepository (Cleveland Clinic Genomic Medicine Institute, Cleveland, OH, USA). LCL cultures were maintained in RPMI 1640 medium supplemented with 20% (by volume) heat inactivated fetal bovine serum (Gibco, Billings, MN, USA) and 1% (by volume) 100U/mL penicillin/streptomycin (Gibco, Billings, MN, USA) at 37°C under humidified atmosphere with 5% CO_2_. All cell lines with assigned unique research code numbers were anonymized and devoid of any participant identifiers.

### Immunofluorescence and image quantification

To induce double strand DNA damage, LCLs were irradiated with 3 Gy of γ-ray during their exponential growth phase at a cell density of about 0.5 million cells/mL. For immunofluorescence (IF) staining, LCLs were captured onto poly-L-lysine (Sigma, St. Louis, MO, USA, cat# P4704) precoated bottom of clear-bottom 96-well plates (Ibidi, Fitchburg, WI, cat# 89626) by centrifugation (110xg for 5 min) at 0.5, 2, 4, 6, 8, and 24 hours after irradiation. Control samples (0 Gy irradiated) were also collected in the same manner. Attached LCLs were washed with PBS, and fixed with 100% methanol for 5 min. After blocking with 2% donkey serum containing PBS for one hour at room temperature, a DNA damage maker, γH2AX, was stained with 1:200 diluted anti-phospho-Histone H2A.X (Ser139) (Millmore Sigma, Burlington, MA cat# 05-636-I) and Alexa-fluor 488 affinity purified Donkey Anti-Mouse IgG (H+L) secondary antibody at 1:1000 dilution (Jackson ImmunoResearch, West Grove, PA, USA). LCL nuclei were stained with 1:1000 diluted Hoechst 33342 (Fisher Waltham, MA, USA, cat# H1399) for DNA content quantification.

The immunofluorescence images of γH2AX and DNA were captured by BioTek BioSpa Live Cell Analysis System (Agilent, Santa Clara, CA, USA) using a 20X objective lens. Images from 9 fields with more than 50 cells per field were collected per condition. We used Fiji (version 2.0.0) software to quantify the obtained images using a previously described method with modification [32]. Firstly, we removed the background fluorescence signals of each image by subtracting the average background image, which was generated by averaging of multiple images of the empty well taken under the same image capturing condition used for imaging the stained cells. Then based on each Hoechst 33342 stained DNA image, mask images that delineate individual nuclear regions, were created by setting a binarization threshold value of DNA images followed by watershed function to separate contacting nuclei. The mask was then applied to the background subtracted γH2AX and DNA fluorescence images to measure the fluorescence intensity of individual nuclei using "analyze particles" function in Fiji.

### Immunoblotting

Whole-cell lysates were made using the Mammalian Protein Extraction Reagent M-PER (Thermo Scientific Pierce, Waltham, MA, USA) supplemented with a cocktail of protease and phosphatase inhibitors (Sigma, St. Louis, MO, USA). Protein content of the lysate was quantified using the BCA Protein Assay Kit (Thermo Scientific Pierce). Proteins in the lysates were separated by SDS-PAGE (Criterion 4–15% gradient gels, BioRad, Hercules, CA, USA) and transferred onto nitrocellulose membranes using the Transblot Turbo system (BioRad). Membranes were blocked with 5% BSA in TBST (tris buffered saline + 0.2% Tween by volume) for an hour at room temperature and then probed with antibodies against PTEN (Millipore, clone 6H2.1, Burlington, MA, USA), phospho-AKT Ser473 (Cell Signaling #4060, Danvers, Massachusetts, USA), total AKT (Cell Signaling #9272), phospho-ERK1/2 (Cell Signaling #9101), total ERK1/2 (Cell Signaling #9102), phospho-Ser139 γH2AX (Millipore 05–636) and GAPDH (Abcam #9484, Waltham, Boston) antibodies which were diluted 1:1000 in blocking buffer. After washing, blots were incubated with secondary antibodies, either HRP-conjugated anti-mouse or anti-rabbit (Promega, 1:5000 dilution in blocking buffer), for 1 hour at room temperature. After 3 times washing with TBS-t, the immunoreactive bands were visualized by incubation with West Pico PLUS Chemiluminescent Substrate (Thermo Scientific Pierce). Blots were scanned digitally and quantified using ImageJ software (NIH). (PI3K-AKT pathway expression results can be checked on S16 Fig)

### Determination of exponential growth rate and carrying capacity of LCL cultures

To analyze LCL proliferation rates during their exponential growth phase, 0.3 million LCL cells in 0.6 mL of medium were initially irradiated with either 3 Gy or 0 Gy (control) and then placed triplicately in a well of a 48-well plate on day 0. We harvested 0.2 ml of the cell suspension from each well and counted viable LCL numbers every day using Vi-CELL BLU Cell Viability Analyzer (Beckman Coulter, Brea, CA, USA). On day 2, the remaining 0.2 mL of cell suspension in each well received 0.4 mL of fresh medium to sustain the LCL cultures. The fresh medium replenishment was repeated every 2 days through the day 5 harvest. Cell count data over 5-day cultures were analyzed by linear regression models on time (day) and log-transformed live cell numbers (million/ml).


log(live cell number)=α time+β

Where α is the exponential transformed live cell growth rate, β is the intercept term.

To determine the carrying capacity for each LCL line, LCLs were seeded in triplicate at various cell densities of 2, 3, 4, 5, 6 million cells per mL in 0.6 mL medium into wells of 48-well plates on day 0. A volume of 200 μl of each culture was processed by Vi-CELL BLU Cell Viability Analyzer to count the cells daily over a duration of 3 days. Once the average viable cell density became lower than the average live cell density minus one standard deviation of the previous day, we stopped counting cells for that specific culture. The carrying capacity for each LCL was determined as the highest peak of viable cell density that exceeded the initial seeding cell densities among the cultures started with various seeding cell densities.

### Mathematical model and statistical analysis

DNA double strand damage repairing experiments were analyzed by the exponential decay formula [33,34]. We fit our data from 2 to 24 hours after irradiation, which only included the exponential decay part and excluded the early DNA damage accumulation phase when experimentally determined γH2AX intensities showed a high degree of variability. We used the exponential decay equation $d=(1-A)e^{-Bt}+C$ to mathematically describe the DNA damage repair kinetics of the LCLs. In the mathematical equation, d represents DNA damage level, A represents a normalization factor; B represents the repair rate; C indicates the baseline DNA damage level of each cell line; and t represents time. Values of A, B and C were determined from the γH2AX time course IF experimental data.

For the mathematical expression of LCL growth curves, we used logistic growth equation $N=\frac{K}{1+e^{-rt}}$ to fit the data. In this formula, N represents the number of viable LCL cells per mL; K indicates the maximized LCL carrying capacity; r is the exponential LCL growth rate and t represents time.

The integrated equation which contains both DNA damage term and cell growth term is a modification of the reaction-diffusion equation partial differential equation (PDE), $\frac{\partial N(d,t)}{\partial t}=(\nabla_{d}(N(d,t)∙f))+rN(1-\frac{N}{K}))$). Here, d represents DNA damage; t represents time; f is the DNA damage repair rate, which will be the first derivative of the above exponential decay equation; and r is the exponential LCL growth rate.

Numerical results of the above PDEs were calculated for every LCL. Then we transferred and normalized the numerical result into 0–8 levels. We averaged the normalized numerical results by specific subgroup, for example PHTS-ASD/DD, to represent the subgroup’s DDR. Therefore, we can easily obtain the difference of DDR between different subgroups by calculating the difference of the corresponding average numerical result. To visualize the DDR difference, we used heatmaps, whose x axis represents time, y axis represents DNA damage levels, and the color represents the difference of cell density. Since x and y are fixed, we can directly identify any differences by color. We applied Kolmogorov–Smirnov test to compare the average numerical results of different PHTS phenotype subgroups at the fixed timepoint, which can help us figure out the DDR difference between the distinct PHTS phenotype subgroups.

All statistical computations were performed using R version 4.2.0. The nonparametric Mann-Whitney test (Wilcoxson test) was used to identify differences between the features of groups clustered by PHTS clinical phenotypes and/or *PTEN* genotypes. P-values < 0.05 were considered statistically significant.

## Supporting information

S1 Fig Box plot of LCL’s maximum cell grow rate without γ irradiation treatment of different PHTS phenotypes and different *PTEN* genotypes. A. Maximum cell growth rate of each LCLs clustering by PHTS phenotypes. Each dot represents one LCL, phenotypes are labeled by dots’ color and shape. B. Maximum cell growth rate of each LCLs clustering by *PTEN* genotype. Each dot represents one LCL, phenotypes are labeled by dots’ color and shape. X-axis represents different clusters, by PHTS phenotypes or *PTEN* genotypes. Y-axis represents maximum cell growth rate (million/(mL·hr)).
(TIFF)

S2 Fig Box plot of LCLs’ carrying capacity of different PHTS phenotypes and different *PTEN* genotypes. A. Carrying capacity of each LCLs clustering by PHTS phenotype. Each dot represents one LCL, phenotypes are also labeled by dots’ color and shape. B. Carrying capacity of each LCLs clustering by *PTEN* genotypes. Each dot represents one LCL, phenotypes are also labeled by dots’ color and shape. X-axis represents different clusters, by PHTS phenotypes or PTEN genotypes. Y-axis represents carrying capacity (million/mL).
(TIFF)

S3 Fig Box plot of maximum cell grow rate without γ irradiation treatment, categorized by samples’ PTEN variant effect. Each dot represents one sample, phenotype represented by color and dot shape. Horizontal line represents internal control, *PTEN* WT LCL’s maximum cell growth rate treated with 0 Gy γ irradiation. Each dot represents one sample’s mtDNA CN value. The upper whisker extends from the hinge to the largest value no further than 1.5 * inter-quartile range (IQR) from the hinge. The lower whisker extends from the hinge to the smallest value at most 1.5 * IQR of the hinge. Data beyond the end of the whiskers are "outlying" points and are plotted individually.
(TIFF)

S4 Fig Box plot of maximum cell grow rate without γ irradiation treatment, categorized by samples’ PTEN variant located region. Each dot represents one sample, phenotype represented by color and dot shape. Horizontal line represents internal control, *PTEN* WT LCL’s maximum cell growth rate treated with 0 Gy γ irradiation. Each dot represents one sample’s mtDNA CN value. The upper whisker extends from the hinge to the largest value no further than 1.5 * inter-quartile range (IQR) from the hinge. The lower whisker extends from the hinge to the smallest value at most 1.5 * IQR of the hinge. Data beyond the end of the whiskers are "outlying" points and are plotted individually.
(TIFF)

S5 Fig Box plot of maximum cell grow rate without γ irradiation treatment, categorized by samples’ *PTEN* variant located within coding region or not. Each dot represents one sample, phenotype represented by color and dot shape. Horizontal line represents internal control, *PTEN* WT LCL’s maximum cell growth rate treated with 0 Gy γ irradiation. Each dot represents one sample’s mtDNA CN value. The upper whisker extends from the hinge to the largest value no further than 1.5 * inter-quartile range (IQR) from the hinge. The lower whisker extends from the hinge to the smallest value at most 1.5 * IQR of the hinge. Data beyond the end of the whiskers are "outlying" points and are plotted individually.
(TIFF)

S6 Fig Box plot of cell’s carrying capacity without γ irradiation treatment, categorized by samples’ *PTEN* variant effect. Each dot represents one sample, phenotype represented by color and dot shape. Horizontal line represents internal control, *PTEN* WT LCL’s carrying capacity. Each dot represents one sample’s mtDNA CN value. The upper whisker extends from the hinge to the largest value no further than 1.5 * inter-quartile range (IQR) from the hinge. The lower whisker extends from the hinge to the smallest value at most 1.5 * IQR of the hinge. Data beyond the end of the whiskers are "outlying" points and are plotted individually.
(TIFF)

S7 Fig Box plot of cell’s carrying capacity without γ irradiation treatment, categorized by samples’ *PTEN* variant located region. Each dot represents one sample, phenotype represented by color and dot shape. Horizontal line represents internal control, *PTEN* WT LCL’s carrying capacity. Each dot represents one sample’s mtDNA CN value. The upper whisker extends from the hinge to the largest value no further than 1.5 * inter-quartile range (IQR) from the hinge. The lower whisker extends from the hinge to the smallest value at most 1.5 * IQR of the hinge. Data beyond the end of the whiskers are "outlying" points and are plotted individually.
(TIFF)

S8 Fig Box plot of cell’s carrying capacity without γ irradiation treatment, categorized by samples’ *PTEN* variant located within coding region or not. Each dot represents one sample, phenotype represented by color and dot shape. Horizontal line represents internal control, *PTEN* WT LCL’s carrying capacity. Each dot represents one sample’s mtDNA CN value. The upper whisker extends from the hinge to the largest value no further than 1.5 * inter-quartile range (IQR) from the hinge. The lower whisker extends from the hinge to the smallest value at most 1.5 * IQR of the hinge. Data beyond the end of the whiskers are "outlying" points and are plotted individually.
(TIFF)

S9 Fig Box plot of maximum cell grow rate after treated with 3 Gy γ irradiation, categorized by samples’ phenotype. Each dot represents one sample, phenotype also represented by color and dot shape. Horizontal line represents internal control, *PTEN* WT LCL’s maximum cell growth rate treated with 3 Gy γ irradiation. Each dot represents one sample’s mtDNA CN value. The upper whisker extends from the hinge to the largest value no further than 1.5 * inter-quartile range (IQR) from the hinge. The lower whisker extends from the hinge to the smallest value at most 1.5 * IQR of the hinge. Data beyond the end of the whiskers are "outlying" points and are plotted individually.
(TIFF)

S10 Fig Box plot of maximum cell grow rate after treated with 3 Gy γ irradiation, categorized by samples’ PTEN genotype. Each dot represents one sample, phenotype represented by color and dot shape. Horizontal line represents internal control, *PTEN* WT LCL’s maximum cell growth rate treated with 3 Gy γ irradiation. Each dot represents one sample’s mtDNA CN value. The upper whisker extends from the hinge to the largest value no further than 1.5 * inter-quartile range (IQR) from the hinge. The lower whisker extends from the hinge to the smallest value at most 1.5 * IQR of the hinge. Data beyond the end of the whiskers are "outlying" points and are plotted individually.
(TIFF)

S11 Fig Box plot of maximum cell grow rate after treated with 3 Gy γ irradiation, categorized by samples’ PTEN variant effect. Each dot represents one sample, phenotype represented by color and dot shape. Horizontal line represents internal control, *PTEN* WT LCL’s maximum cell growth rate treated with 3 Gy γ irradiation. Each dot represents one sample’s mtDNA CN value. The upper whisker extends from the hinge to the largest value no further than 1.5 * inter-quartile range (IQR) from the hinge. The lower whisker extends from the hinge to the smallest value at most 1.5 * IQR of the hinge. Data beyond the end of the whiskers are "outlying" points and are plotted individually.
(TIFF)

S12 Fig Box plot of maximum cell grow rate difference before and after treated with 3 Gy γ irradiation, categorized by samples’ PTEN variant location. Each dot represents one sample, phenotype represented by color and dot shape. Horizontal line represents internal control, *PTEN* WT LCL’s maximum cell growth rate change treated with and without 3 Gy γ irradiation. Each dot represents one sample’s mtDNA CN value. The upper whisker extends from the hinge to the largest value no further than 1.5 * inter-quartile range (IQR) from the hinge. The lower whisker extends from the hinge to the smallest value at most 1.5 * IQR of the hinge. Data beyond the end of the whiskers are "outlying" points and are plotted individually.
(TIFF)

S13 Fig Box plot of maximum cell grow rate difference before and after treated with 3 Gy γ irradiation, categorized by samples’ PTEN variant category. Each dot represents one sample, phenotype represented by color and dot shape. Horizontal line represents internal control, *PTEN* WT LCL’s maximum cell growth rate change treated with and without 3 Gy γ irradiation. Each dot represents one sample’s mtDNA CN value. The upper whisker extends from the hinge to the largest value no further than 1.5 * inter-quartile range (IQR) from the hinge. The lower whisker extends from the hinge to the smallest value at most 1.5 * IQR of the hinge. Data beyond the end of the whiskers are "outlying" points and are plotted individually.
(TIFF)

S14 Fig Box plot of maximum cell grow rate difference before and after treated with 3 Gy γ irradiation, categorized by samples’ PTEN variant effect. Each dot represents one sample, phenotype represented by color and dot shape. Horizontal line represents internal control, *PTEN* WT LCL’s maximum cell growth rate change treated with and without 3 Gy γ irradiation. Each dot represents one sample’s mtDNA CN value. The upper whisker extends from the hinge to the largest value no further than 1.5 * inter-quartile range (IQR) from the hinge. The lower whisker extends from the hinge to the smallest value at most 1.5 * IQR of the hinge. Data beyond the end of the whiskers are "outlying" points and are plotted individually.
(TIFF)

S15 Fig Box plot of maximum cell grow rate difference before and after treated with 3 Gy γ irradiation, categorized by samples’ PTEN genotype. Each dot represents one sample, phenotype represented by color and dot shape. Horizontal line represents internal control, *PTEN* WT LCL’s maximum cell growth rate change treated with and without 3 Gy γ irradiation. Each dot represents one sample’s mtDNA CN value. The upper whisker extends from the hinge to the largest value no further than 1.5 * inter-quartile range (IQR) from the hinge. The lower whisker extends from the hinge to the smallest value at most 1.5 * IQR of the hinge. Data beyond the end of the whiskers are "outlying" points and are plotted individually.
(TIFF)

S16 Fig Western blots and quantified results of PTEN canonical pathway expression of representative LCLs with different *PTEN* genotypes. (TIFF)

S17 Fig Euclid distance between average numerical results from different subgroups of PHTS phenotype and/or *PTEN* genotype effect. (TIFF)
