# Supplementary figures and images for: Quantitative evaluation of DNA damage repair dynamics to elucidate predictors of autism vs. cancer in individuals with germline PTEN variants
Source: PLoS Comput Biol. 2024 Oct 2;20(10):e1012449. doi: 10.1371/journal.pcbi.1012449 (PMC11472915; doi:10.1371/journal.pcbi.1012449)

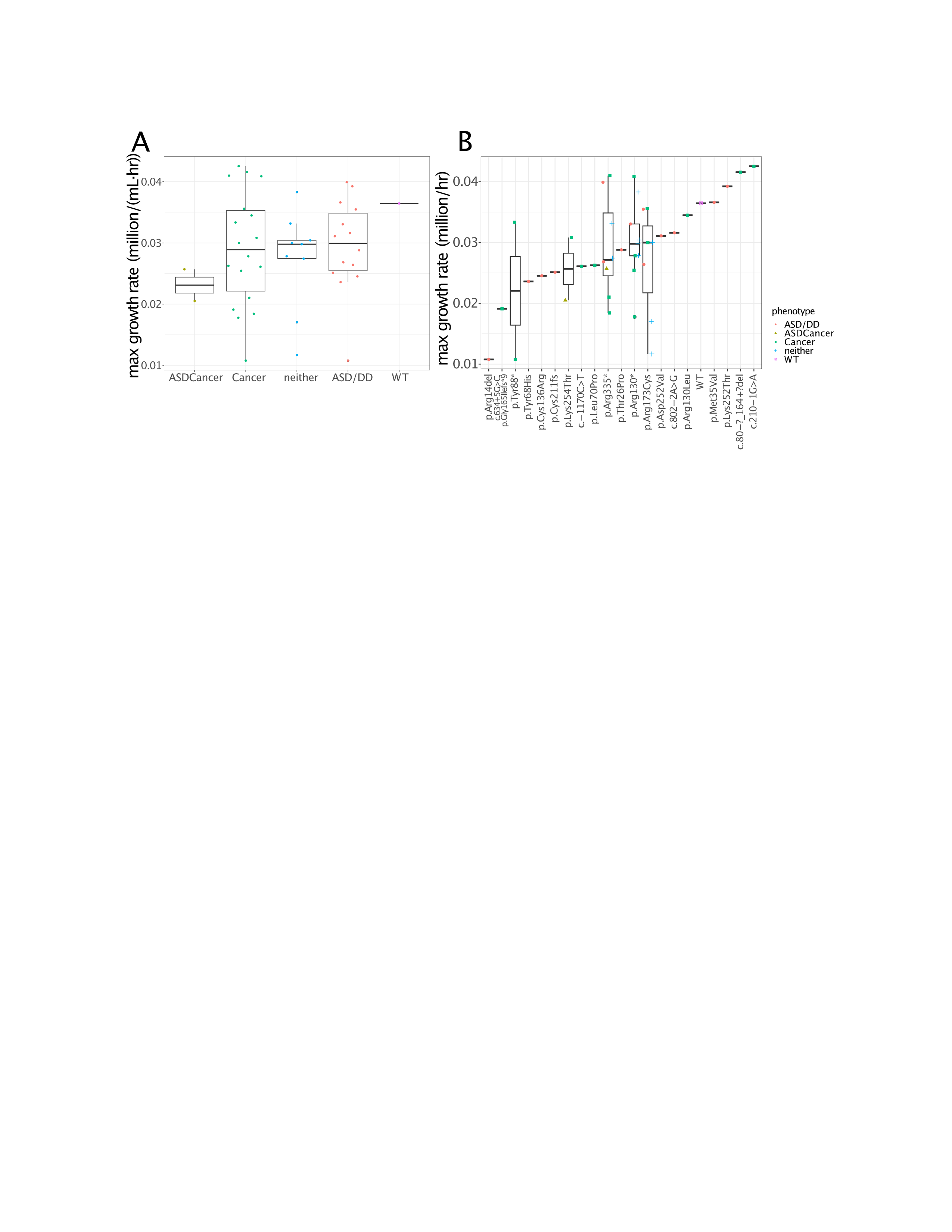

Supplement: S1 Fig — A. Maximum cell growth rate of each LCLs clustering by PHTS phenotypes. Each dot represents one LCL, phenotypes are labeled by dots’ color and shape. B. Maximum cell growth rate of each LCLs clustering by PTEN genotype. Each dot represents one LCL, phenotypes are labeled by dots’ color and shape. X-axis represents different clusters, by PHTS phenotypes or PTEN genotypes. Y-axis represents maximum cell growth rate (million/(mL·hr)). (TIFF) [file pcbi.1012449.s001.tiff]

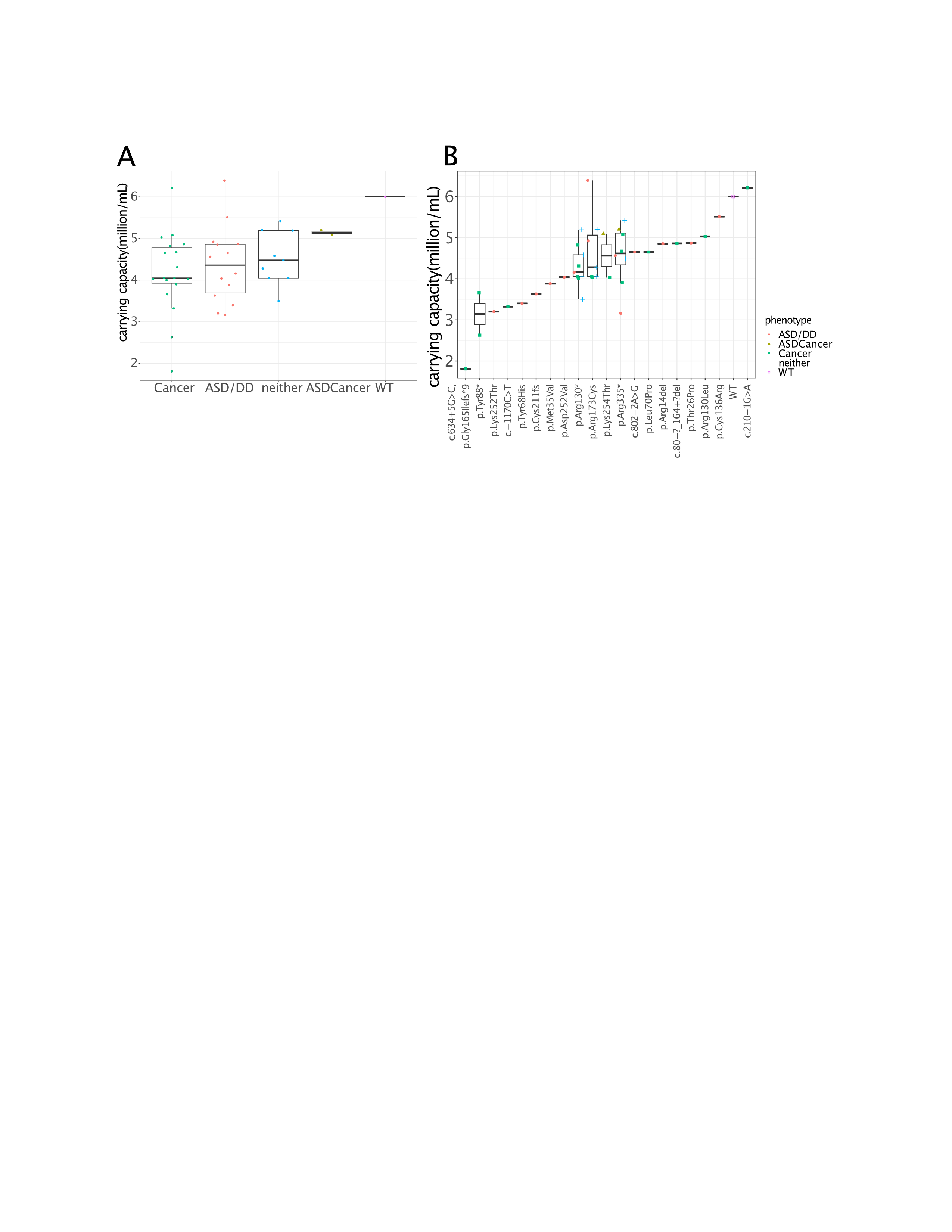

Supplement: S2 Fig — A. Carrying capacity of each LCLs clustering by PHTS phenotype. Each dot represents one LCL, phenotypes are also labeled by dots’ color and shape. B. Carrying capacity of each LCLs clustering by PTEN genotypes. Each dot represents one LCL, phenotypes are also labeled by dots’ color and shape. X-axis represents different clusters, by PHTS phenotypes or PTEN genotypes. Y-axis represents carrying capacity (million/mL). (TIFF) [file pcbi.1012449.s002.tiff]

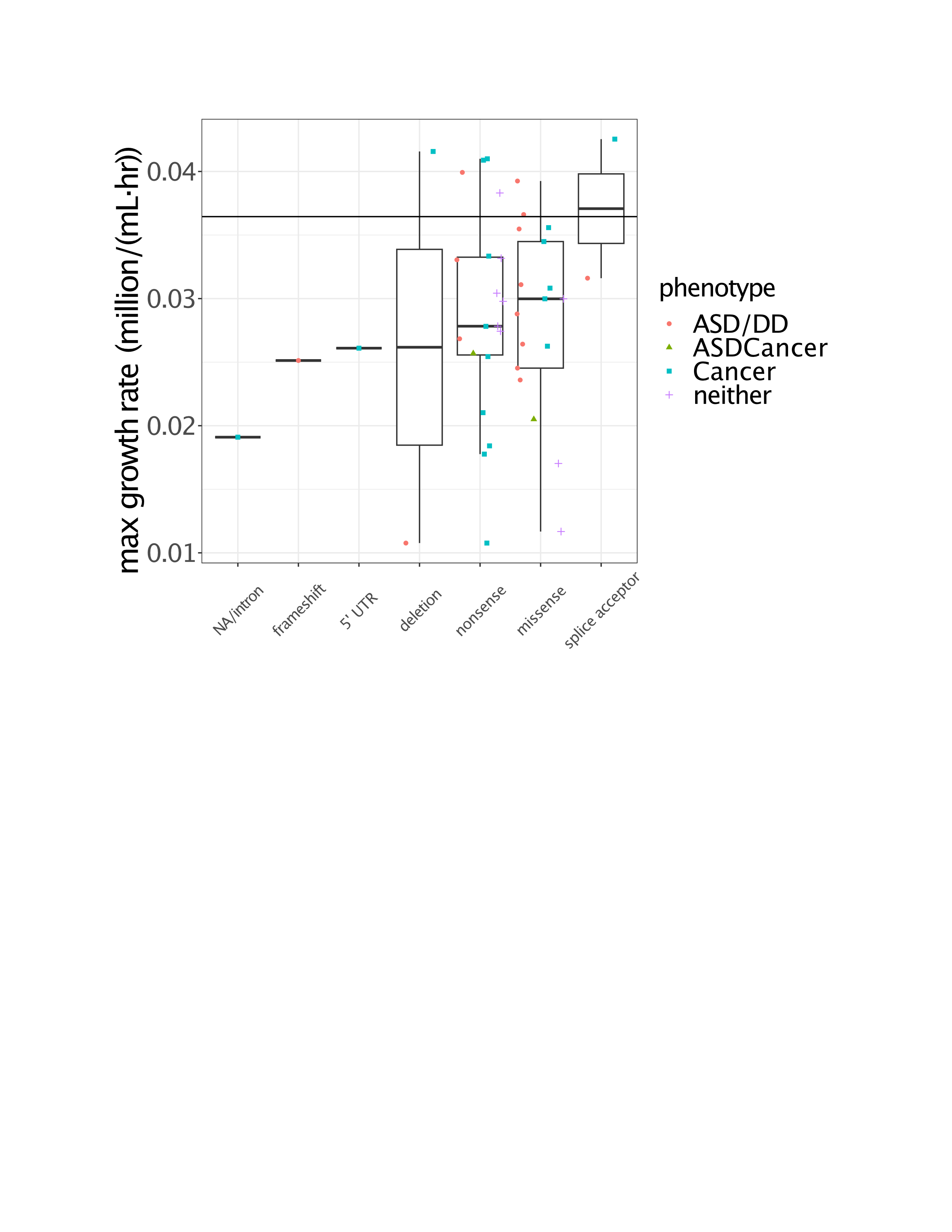

Supplement: S3 Fig — Each dot represents one sample, phenotype represented by color and dot shape. Horizontal line represents internal control, PTEN WT LCL’s maximum cell growth rate treated with 0 Gy γ irradiation. Each dot represents one sample’s mtDNA CN value. The upper whisker extends from the hinge to the largest value no further than 1.5 * inter-quartile range (IQR) from the hinge. The lower whisker extends from the hinge to the smallest value at most 1.5 * IQR of the hinge. Data beyond the end of the whiskers are "outlying" points and are plotted individually. (TIFF) [file pcbi.1012449.s003.tiff]

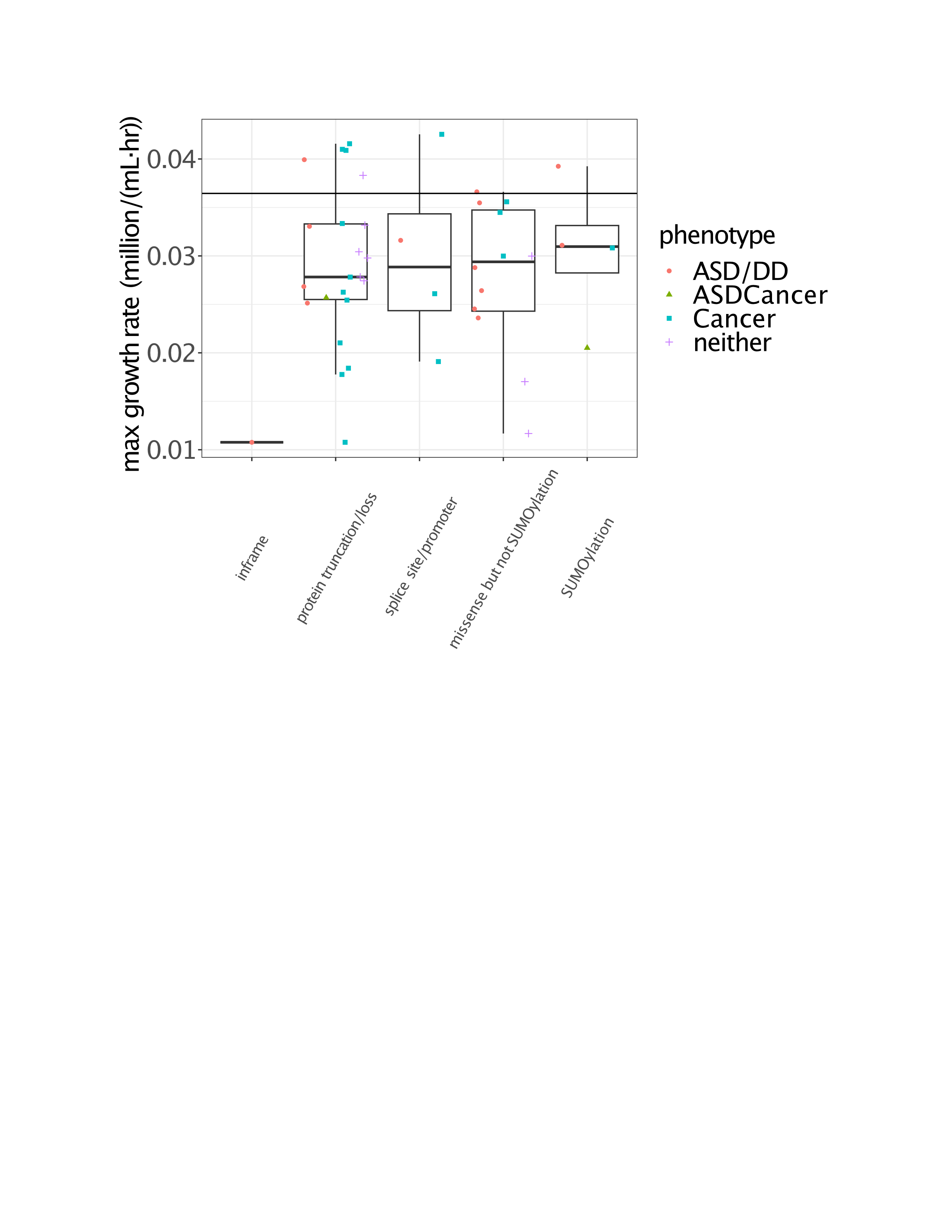

Supplement: S4 Fig — Each dot represents one sample, phenotype represented by color and dot shape. Horizontal line represents internal control, PTEN WT LCL’s maximum cell growth rate treated with 0 Gy γ irradiation. Each dot represents one sample’s mtDNA CN value. The upper whisker extends from the hinge to the largest value no further than 1.5 * inter-quartile range (IQR) from the hinge. The lower whisker extends from the hinge to the smallest value at most 1.5 * IQR of the hinge. Data beyond the end of the whiskers are "outlying" points and are plotted individually. (TIFF) [file pcbi.1012449.s004.tiff]

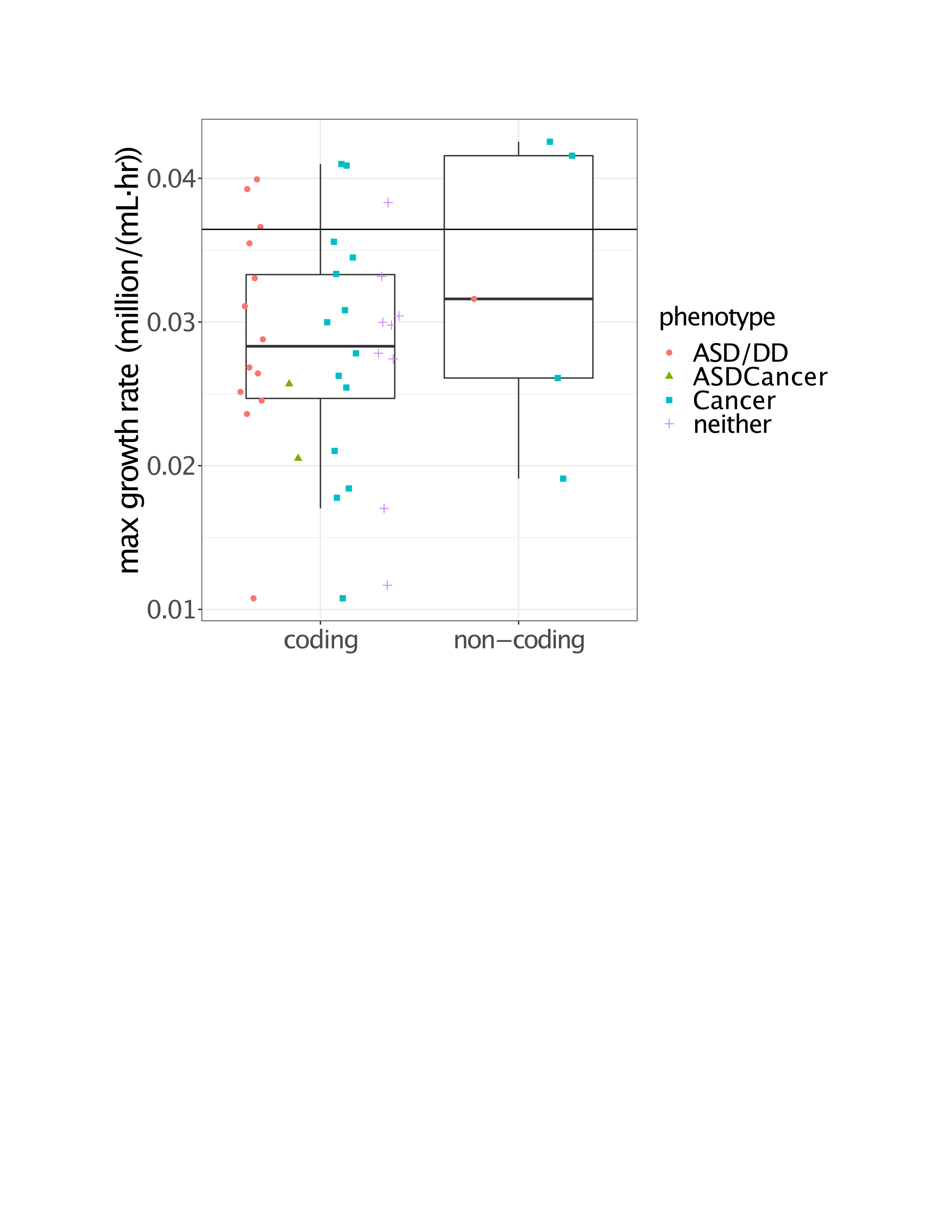

Supplement: S5 Fig — Each dot represents one sample, phenotype represented by color and dot shape. Horizontal line represents internal control, PTEN WT LCL’s maximum cell growth rate treated with 0 Gy γ irradiation. Each dot represents one sample’s mtDNA CN value. The upper whisker extends from the hinge to the largest value no further than 1.5 * inter-quartile range (IQR) from the hinge. The lower whisker extends from the hinge to the smallest value at most 1.5 * IQR of the hinge. Data beyond the end of the whiskers are "outlying" points and are plotted individually. (TIFF) [file pcbi.1012449.s005.tiff]

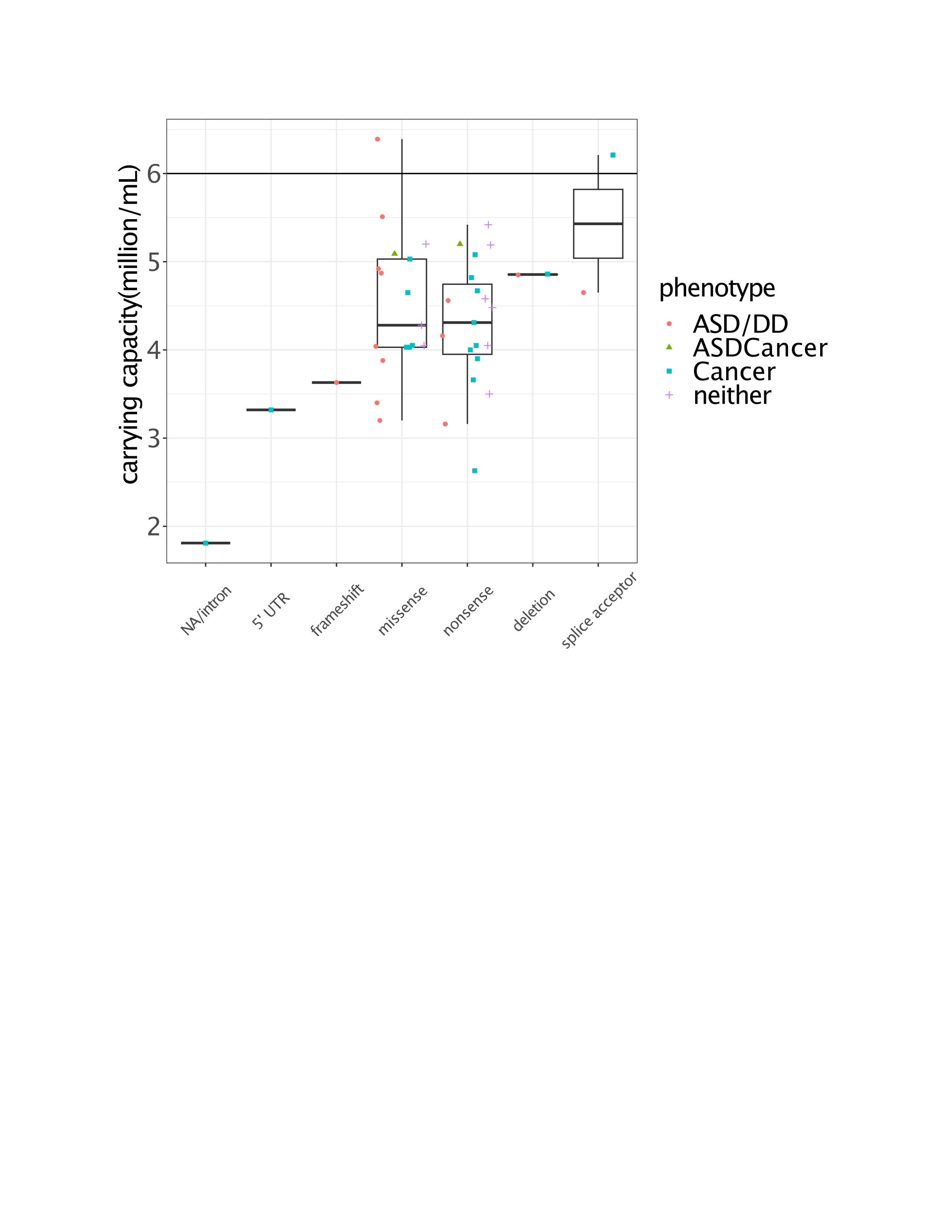

Supplement: S6 Fig — Each dot represents one sample, phenotype represented by color and dot shape. Horizontal line represents internal control, PTEN WT LCL’s carrying capacity. Each dot represents one sample’s mtDNA CN value. The upper whisker extends from the hinge to the largest value no further than 1.5 * inter-quartile range (IQR) from the hinge. The lower whisker extends from the hinge to the smallest value at most 1.5 * IQR of the hinge. Data beyond the end of the whiskers are "outlying" points and are plotted individually. (TIFF) [file pcbi.1012449.s006.tiff]

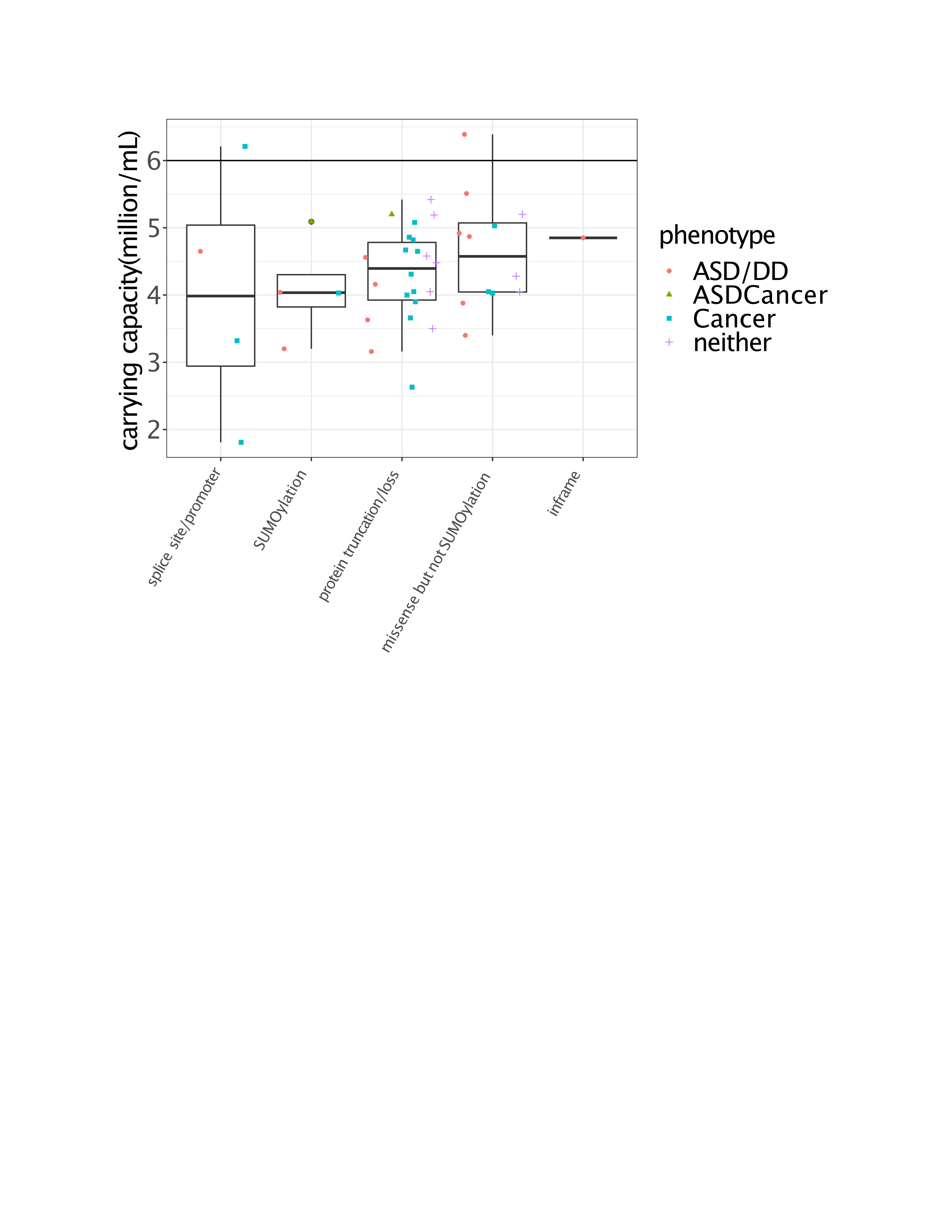

Supplement: S7 Fig — Each dot represents one sample, phenotype represented by color and dot shape. Horizontal line represents internal control, PTEN WT LCL’s carrying capacity. Each dot represents one sample’s mtDNA CN value. The upper whisker extends from the hinge to the largest value no further than 1.5 * inter-quartile range (IQR) from the hinge. The lower whisker extends from the hinge to the smallest value at most 1.5 * IQR of the hinge. Data beyond the end of the whiskers are "outlying" points and are plotted individually. (TIFF) [file pcbi.1012449.s007.tiff]

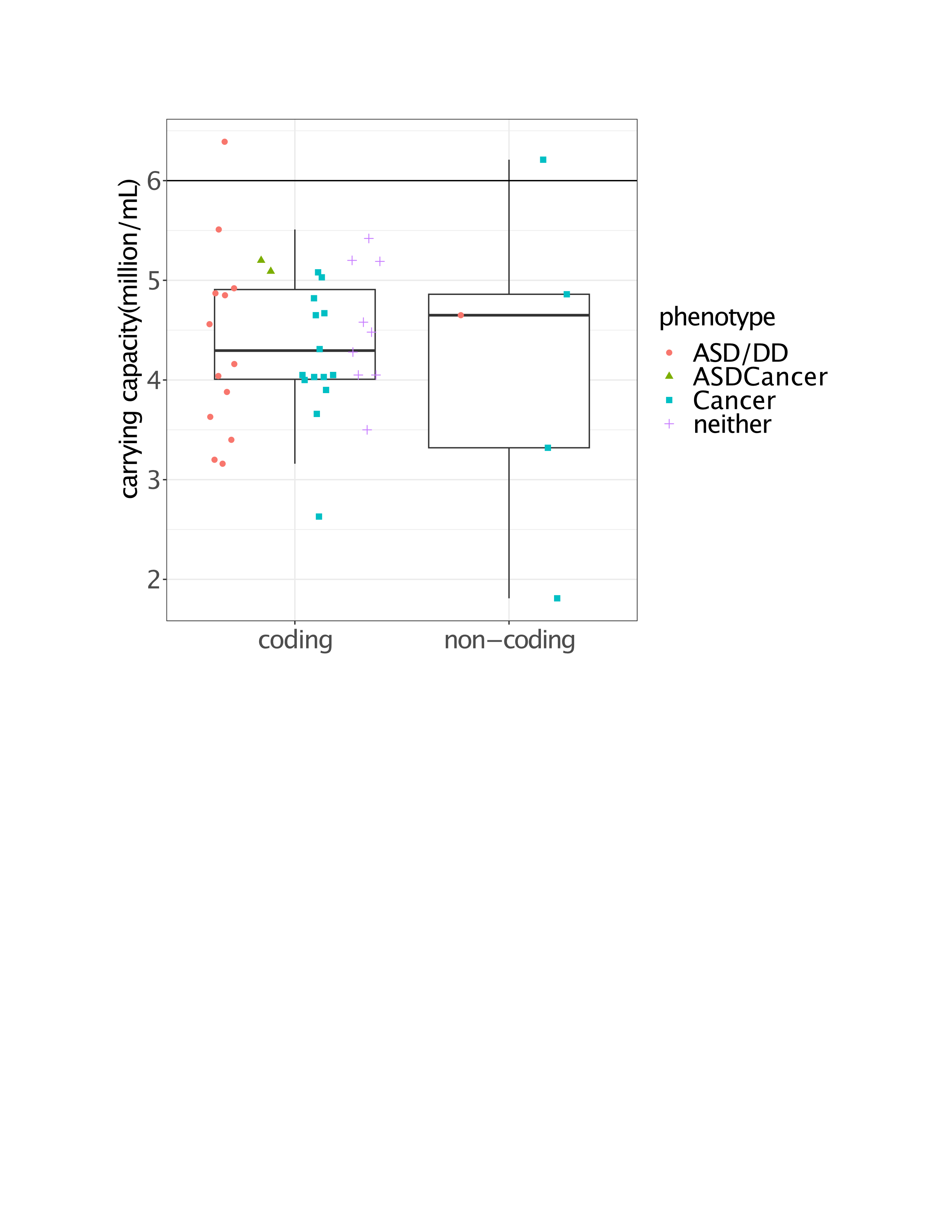

Supplement: S8 Fig — Each dot represents one sample, phenotype represented by color and dot shape. Horizontal line represents internal control, PTEN WT LCL’s carrying capacity. Each dot represents one sample’s mtDNA CN value. The upper whisker extends from the hinge to the largest value no further than 1.5 * inter-quartile range (IQR) from the hinge. The lower whisker extends from the hinge to the smallest value at most 1.5 * IQR of the hinge. Data beyond the end of the whiskers are "outlying" points and are plotted individually. (TIFF) [file pcbi.1012449.s008.tiff]

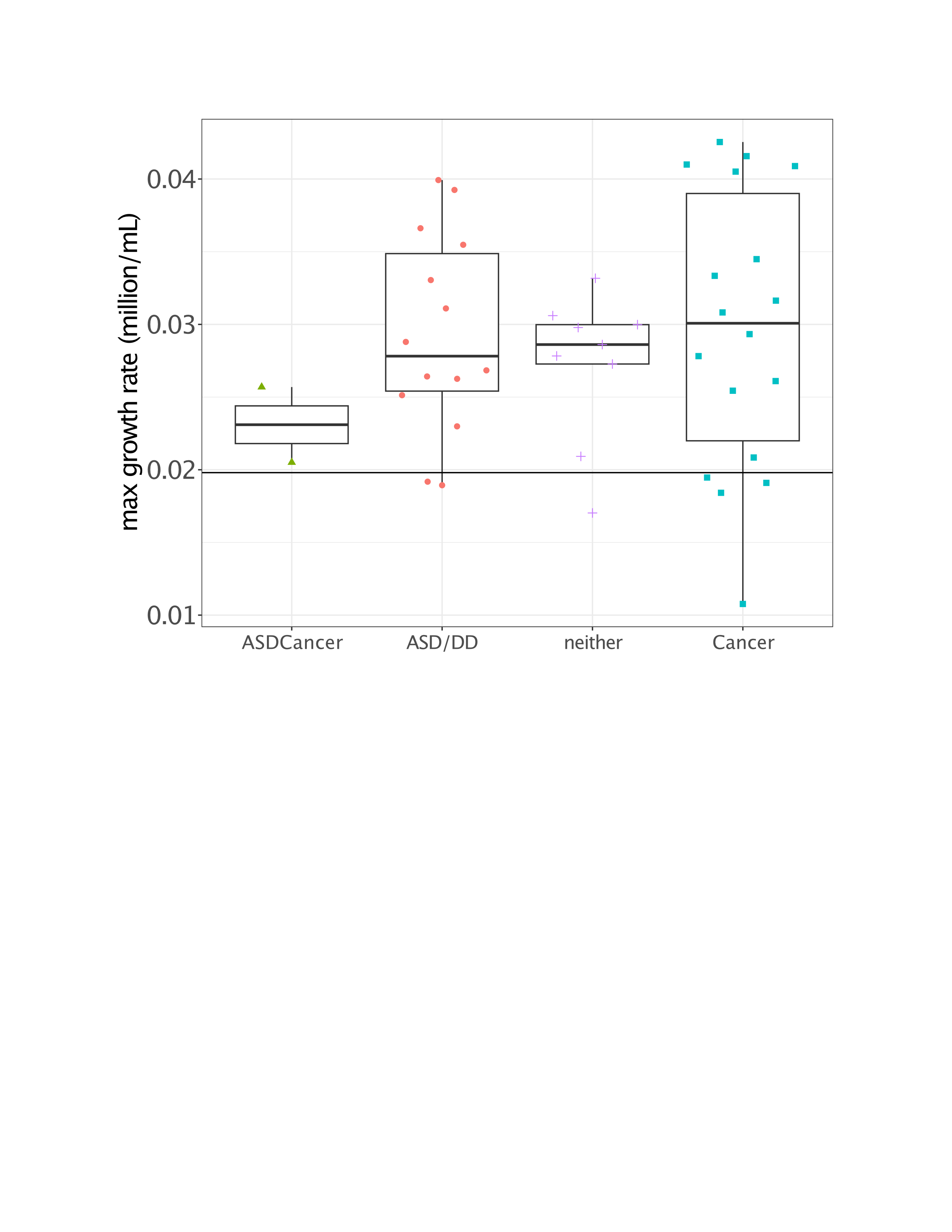

Supplement: S9 Fig — Each dot represents one sample, phenotype also represented by color and dot shape. Horizontal line represents internal control, PTEN WT LCL’s maximum cell growth rate treated with 3 Gy γ irradiation. Each dot represents one sample’s mtDNA CN value. The upper whisker extends from the hinge to the largest value no further than 1.5 * inter-quartile range (IQR) from the hinge. The lower whisker extends from the hinge to the smallest value at most 1.5 * IQR of the hinge. Data beyond the end of the whiskers are "outlying" points and are plotted individually. (TIFF) [file pcbi.1012449.s009.tiff]

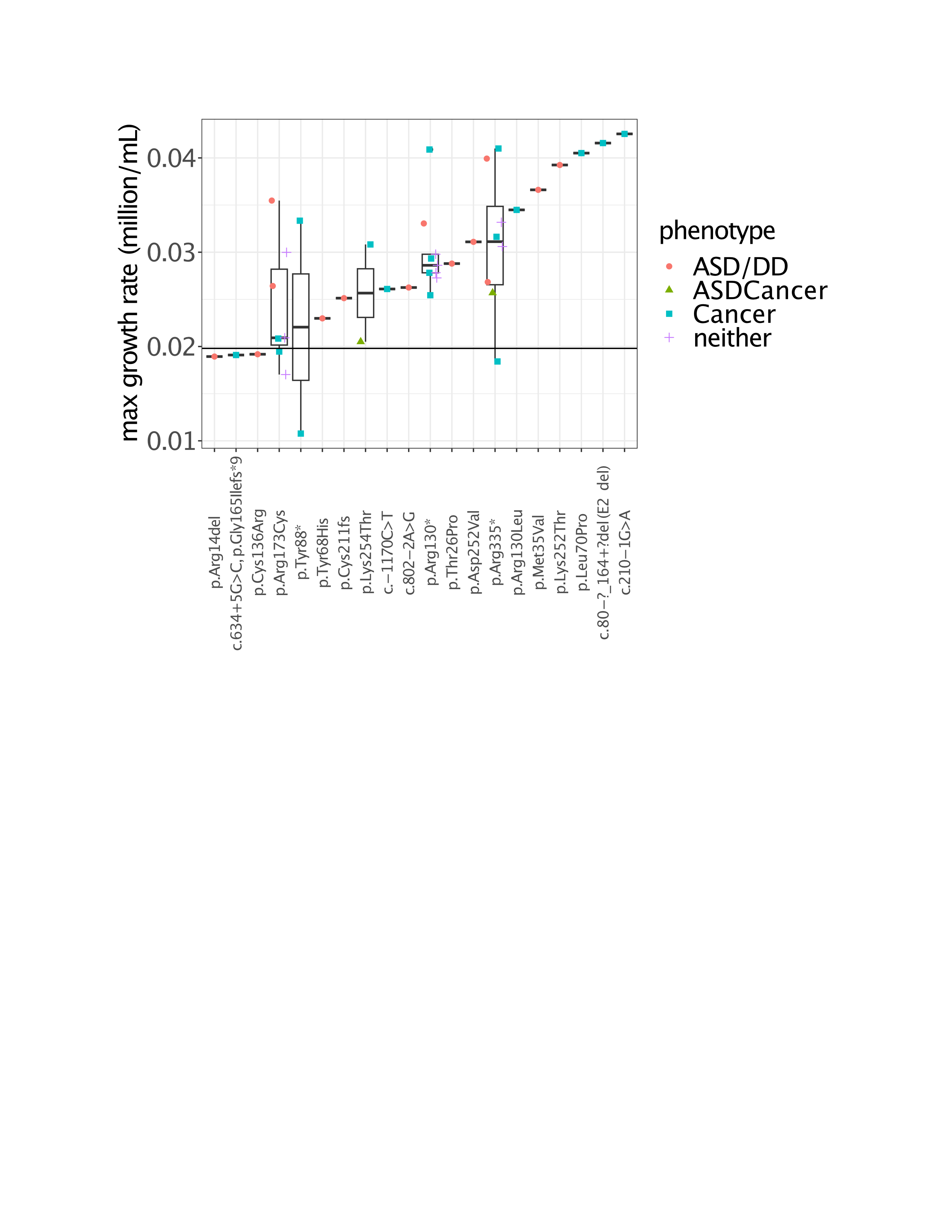

Supplement: S10 Fig — Each dot represents one sample, phenotype represented by color and dot shape. Horizontal line represents internal control, PTEN WT LCL’s maximum cell growth rate treated with 3 Gy γ irradiation. Each dot represents one sample’s mtDNA CN value. The upper whisker extends from the hinge to the largest value no further than 1.5 * inter-quartile range (IQR) from the hinge. The lower whisker extends from the hinge to the smallest value at most 1.5 * IQR of the hinge. Data beyond the end of the whiskers are "outlying" points and are plotted individually. (TIFF) [file pcbi.1012449.s010.tiff]

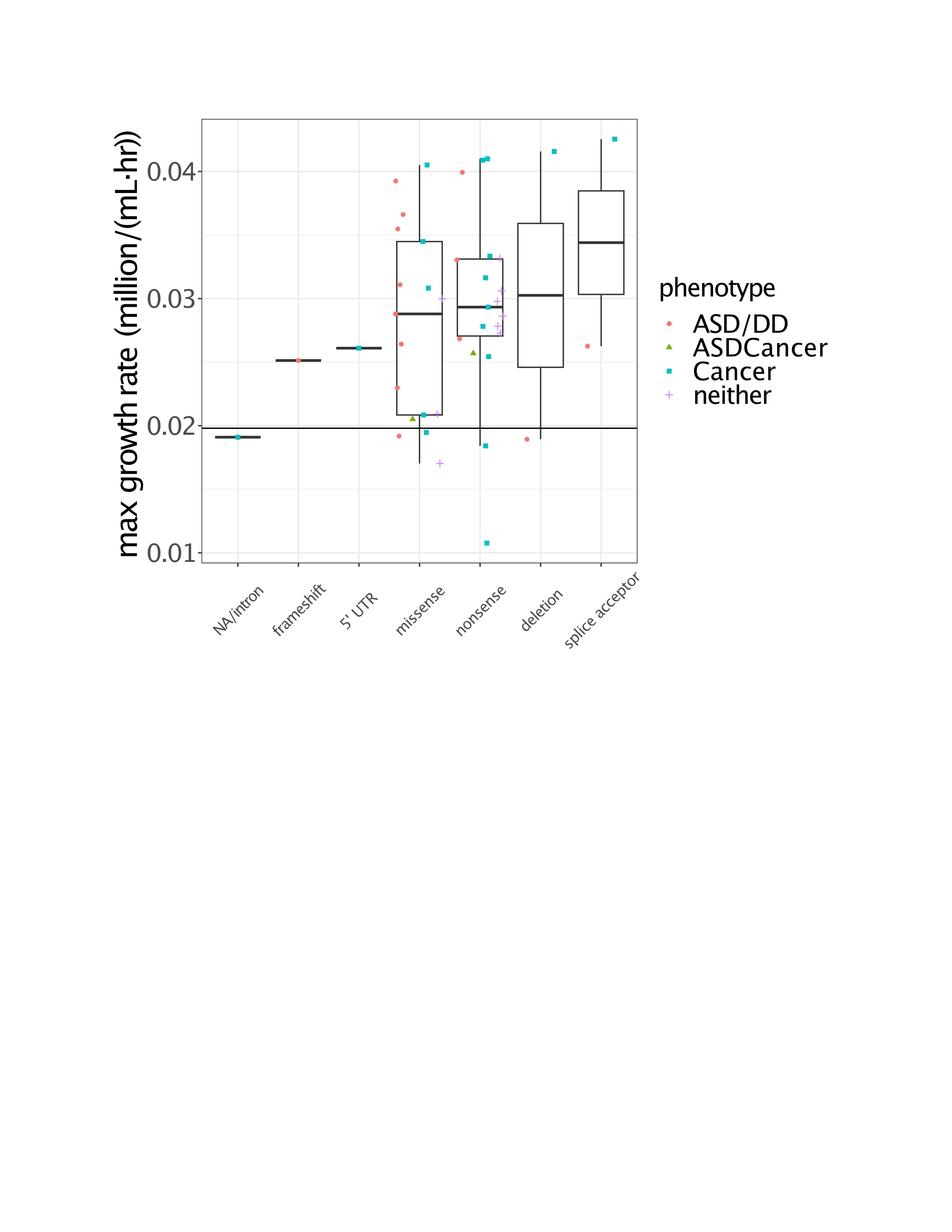

Supplement: S11 Fig — Each dot represents one sample, phenotype represented by color and dot shape. Horizontal line represents internal control, PTEN WT LCL’s maximum cell growth rate treated with 3 Gy γ irradiation. Each dot represents one sample’s mtDNA CN value. The upper whisker extends from the hinge to the largest value no further than 1.5 * inter-quartile range (IQR) from the hinge. The lower whisker extends from the hinge to the smallest value at most 1.5 * IQR of the hinge. Data beyond the end of the whiskers are "outlying" points and are plotted individually. (TIFF) [file pcbi.1012449.s011.tiff]

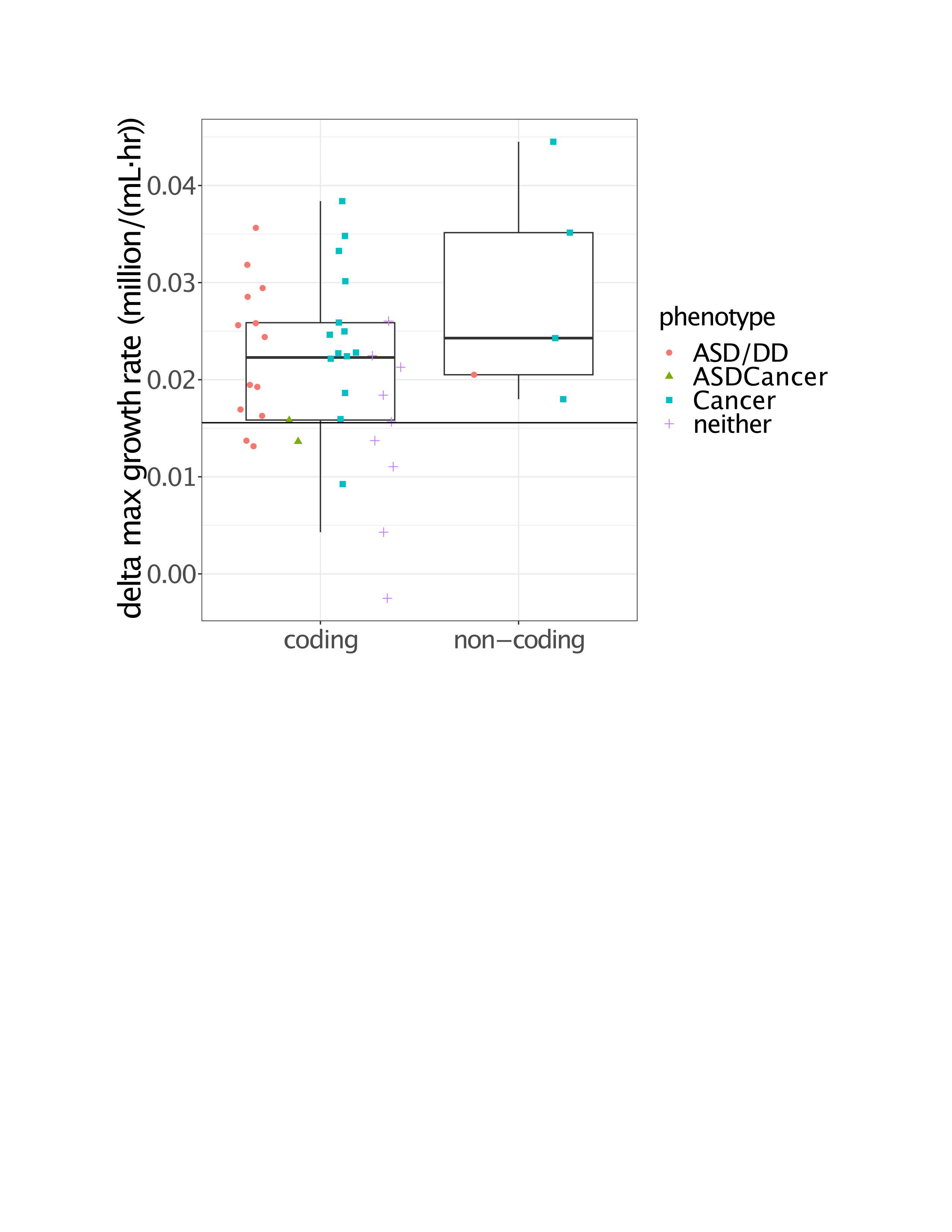

Supplement: S12 Fig — Each dot represents one sample, phenotype represented by color and dot shape. Horizontal line represents internal control, PTEN WT LCL’s maximum cell growth rate change treated with and without 3 Gy γ irradiation. Each dot represents one sample’s mtDNA CN value. The upper whisker extends from the hinge to the largest value no further than 1.5 * inter-quartile range (IQR) from the hinge. The lower whisker extends from the hinge to the smallest value at most 1.5 * IQR of the hinge. Data beyond the end of the whiskers are "outlying" points and are plotted individually. (TIFF) [file pcbi.1012449.s012.tiff]

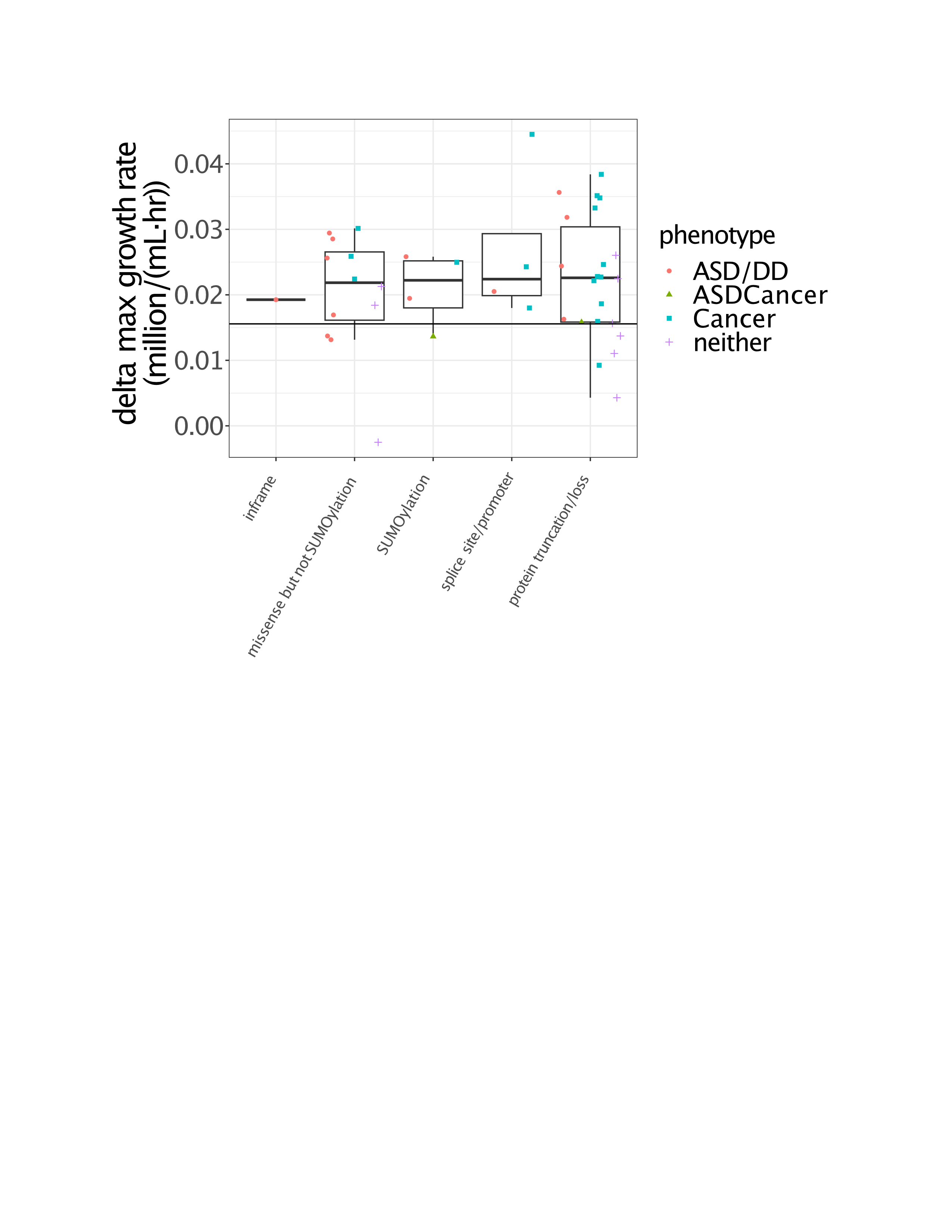

Supplement: S13 Fig — Each dot represents one sample, phenotype represented by color and dot shape. Horizontal line represents internal control, PTEN WT LCL’s maximum cell growth rate change treated with and without 3 Gy γ irradiation. Each dot represents one sample’s mtDNA CN value. The upper whisker extends from the hinge to the largest value no further than 1.5 * inter-quartile range (IQR) from the hinge. The lower whisker extends from the hinge to the smallest value at most 1.5 * IQR of the hinge. Data beyond the end of the whiskers are "outlying" points and are plotted individually. (TIFF) [file pcbi.1012449.s013.tiff]

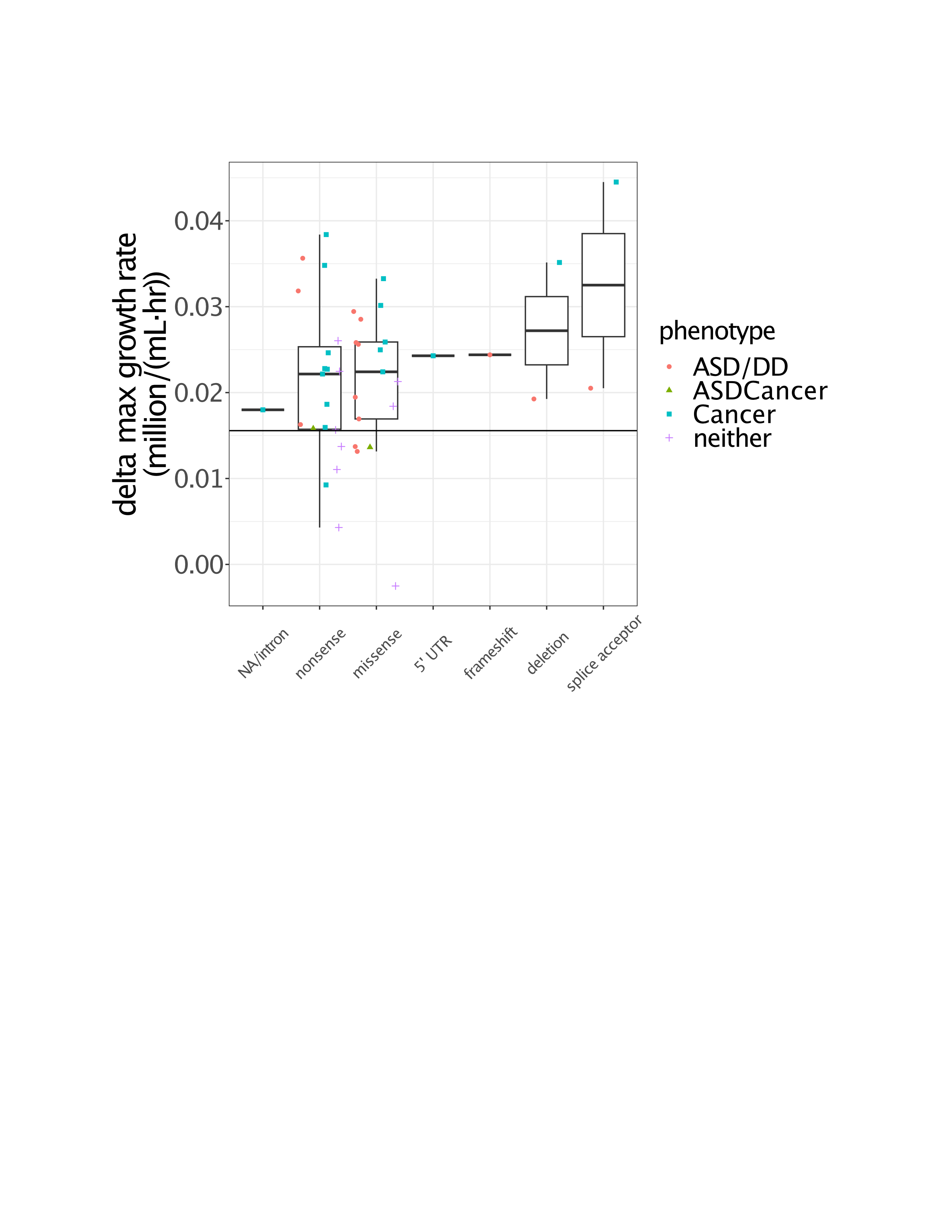

Supplement: S14 Fig — Each dot represents one sample, phenotype represented by color and dot shape. Horizontal line represents internal control, PTEN WT LCL’s maximum cell growth rate change treated with and without 3 Gy γ irradiation. Each dot represents one sample’s mtDNA CN value. The upper whisker extends from the hinge to the largest value no further than 1.5 * inter-quartile range (IQR) from the hinge. The lower whisker extends from the hinge to the smallest value at most 1.5 * IQR of the hinge. Data beyond the end of the whiskers are "outlying" points and are plotted individually. (TIFF) [file pcbi.1012449.s014.tiff]

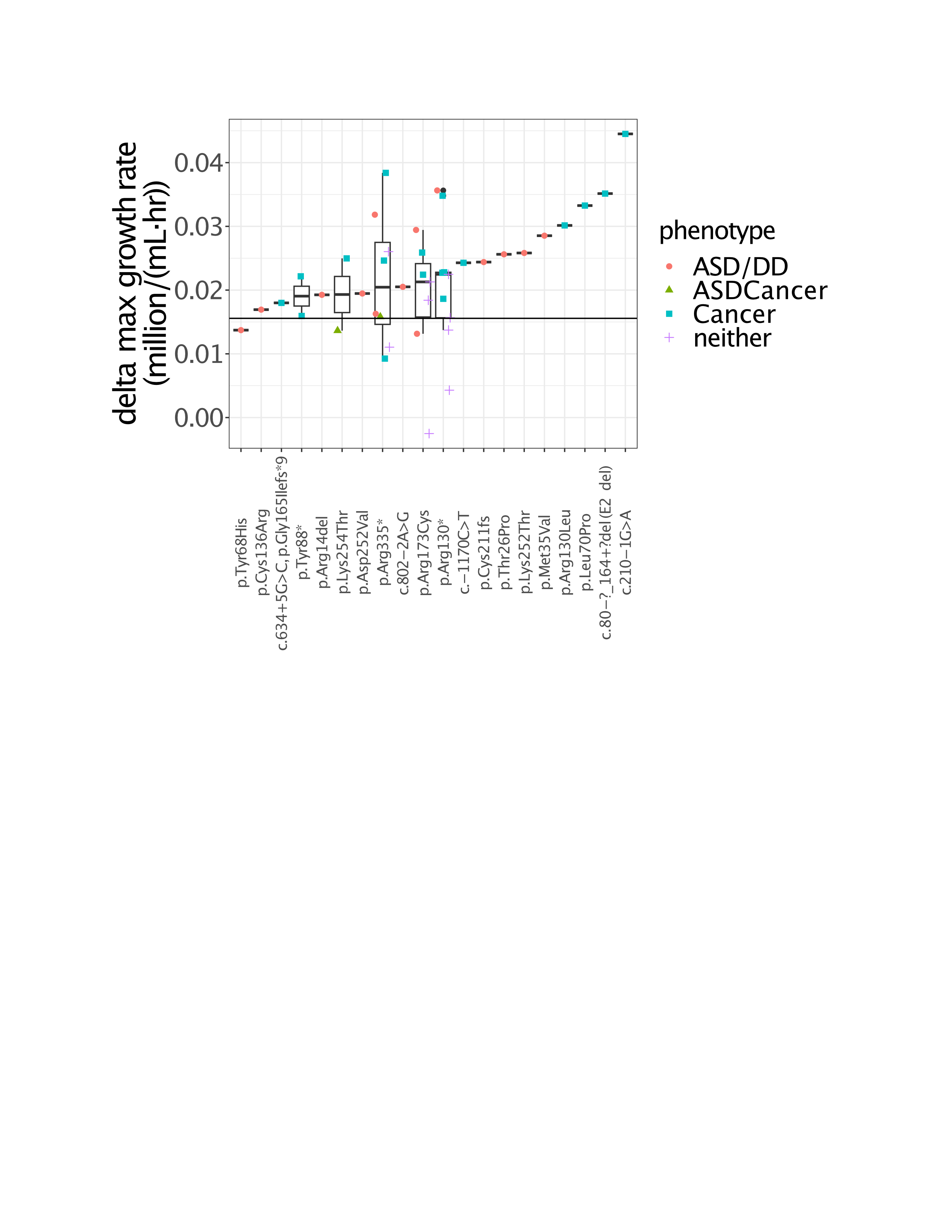

Supplement: S15 Fig — Each dot represents one sample, phenotype represented by color and dot shape. Horizontal line represents internal control, PTEN WT LCL’s maximum cell growth rate change treated with and without 3 Gy γ irradiation. Each dot represents one sample’s mtDNA CN value. The upper whisker extends from the hinge to the largest value no further than 1.5 * inter-quartile range (IQR) from the hinge. The lower whisker extends from the hinge to the smallest value at most 1.5 * IQR of the hinge. Data beyond the end of the whiskers are "outlying" points and are plotted individually. (TIFF) [file pcbi.1012449.s015.tiff]

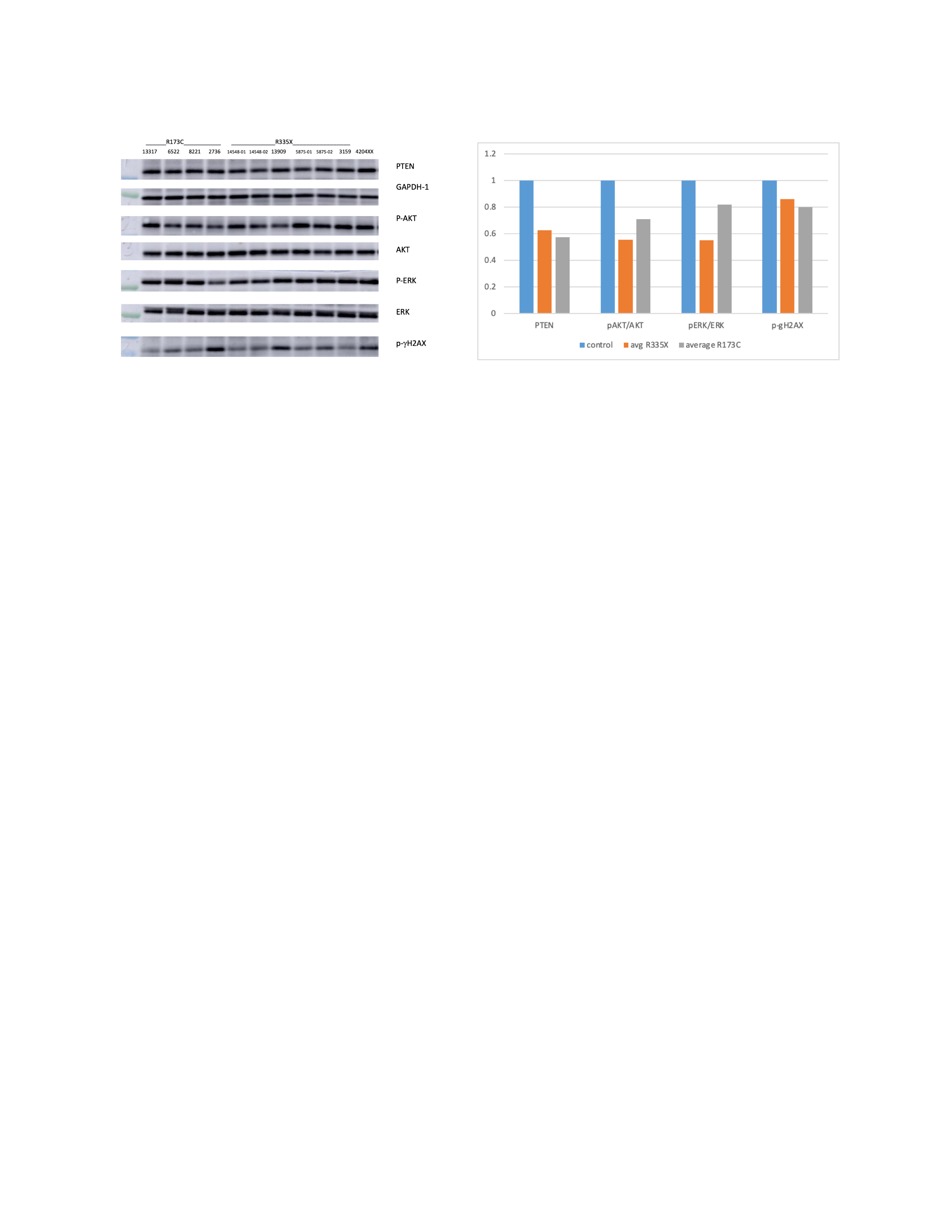

Supplement: S16 Fig — (TIFF) [file pcbi.1012449.s016.tiff]

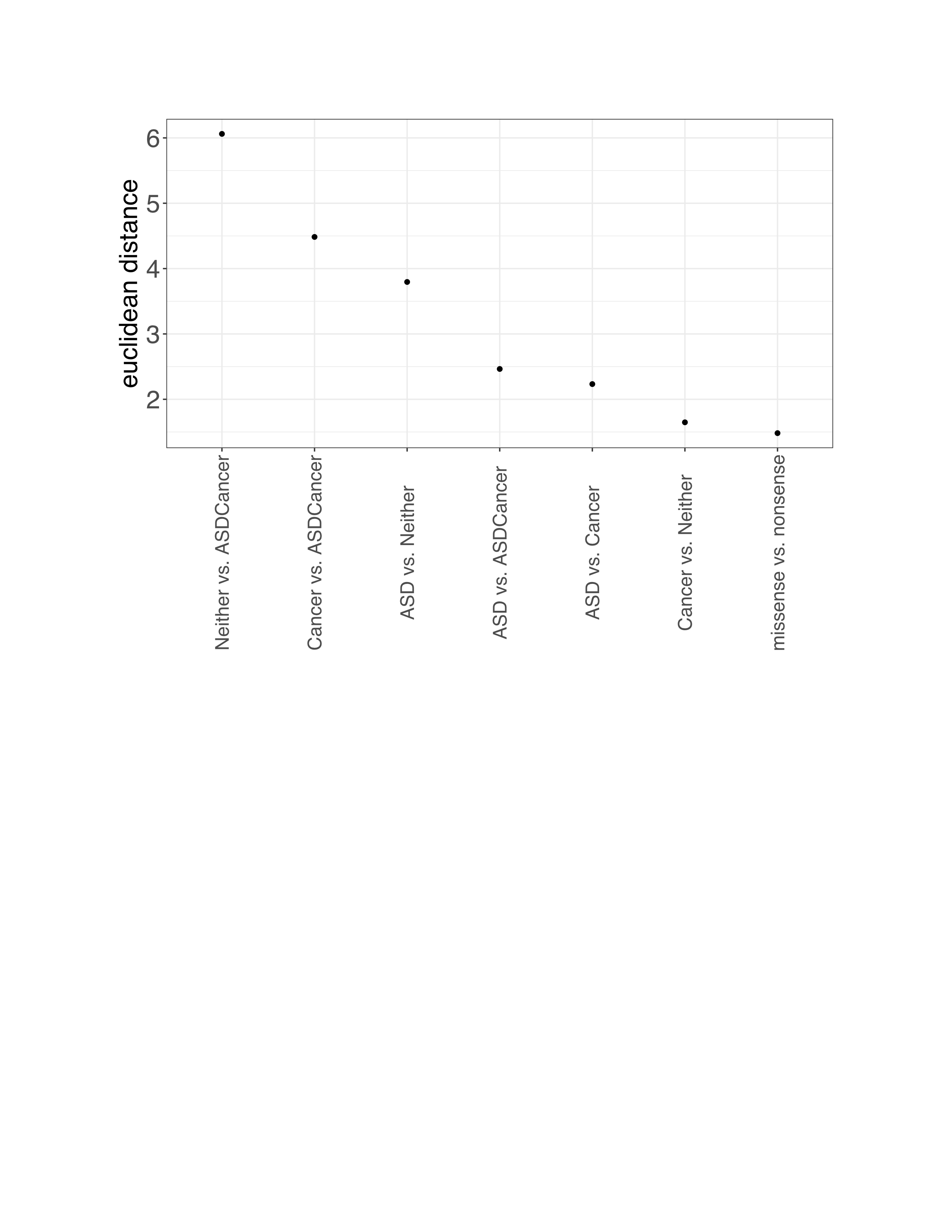

Supplement: S17 Fig — (TIFF) [file pcbi.1012449.s017.tiff]
